# Doxorubicin-induced cardiomyopathy associated with inhibition of autophagic degradation process and defects in mitochondrial respiration

**DOI:** 10.1038/s41598-018-37862-3

**Published:** 2019-02-14

**Authors:** Chowdhury S. Abdullah, Shafiul Alam, Richa Aishwarya, Sumitra Miriyala, Mohammad Alfrad Nobel Bhuiyan, Manikandan Panchatcharam, Christopher B. Pattillo, A. Wayne Orr, Junichi Sadoshima, Joseph A. Hill, Md. Shenuarin Bhuiyan

**Affiliations:** 1 Department of Pathology and Translational Pathobiology, Louisiana State University Health Sciences Center-Shreveport, Shreveport, LA 71103 USA; 2 Department of Molecular and Cellular Physiology, Louisiana State University Health Sciences Center-Shreveport, Shreveport, LA 71103 USA; 3 Department of Cellular Biology and Anatomy, Louisiana State University Health Sciences Center-Shreveport, Shreveport, LA 71103 USA; 4 Division of Biostatistics and Epidemiology, Cincinnati Children’s Hospital, Cincinnati, OH 45229 USA; 5 Department of Cell Biology and Molecular Medicine, Rutgers New Jersey Medical School, Newark, NJ 07103 USA; 6 Department of Internal Medicine (Cardiology), UT Southwestern Medical Center, Dallas, TX 75390 USA; 7 Department of Molecular Biology, UT Southwestern Medical Center, UT Southwestern Medical Center, Dallas, TX 75390 USA

## Abstract

Doxorubicin (Dox) is a highly effective anticancer drug but cause acute ventricular dysfunction, and also induce late-onset cardiomyopathy and heart failure. Despite extensive studies, the pathogenic sequelae leading to the progression of Dox-associated cardiomyopathy remains unknown. We assessed temporal changes in autophagy, mitochondrial dynamics, and bioenergetics in mouse models of acute and chronic Dox-cardiomyopathy. Time course study of acute Dox-treatment showed accumulation of LC3B II in heart lysates. Autophagy flux assays confirmed that the Dox-induced accumulation of autophagosomes occurs due to blockage of the lysosomal degradation process. Dox-induced autophagosomes and autolysosome accumulation were confirmed *in vivo* by using GFP-LC3 and mRFP-GFP-LC3 transgenic (Tg) mice. Mitochondria isolated from acute Dox-treated hearts showed significant suppression of oxygen consumption rate (OCR). Chronic Dox-cardiotoxicity also exhibited time-dependent accumulation of LC3B II levels and increased accumulation of green puncta in GFP-LC3 Tg hearts. Mitochondria isolated from chronic Dox-treated hearts also showed significant suppression of mitochondrial OCR. The *in vivo* impairment of autophagic degradation process and mitochondrial dysfunction data were confirmed *in vitro* using cultured neonatal cardiomyocytes. Both acute and chronic Dox-associated cardiomyopathy involves a multifocal disease process resulting from autophagosomes and autolysosomes accumulation, altered expression of mitochondrial dynamics and oxidative phosphorylation regulatory proteins, and mitochondrial respiratory dysfunction.

## Introduction

Since their discovery more than 50 years ago, anthracyclines (e.g. Doxorubicin) have become the mainstay for the treatment of many childhood and adult malignancies^1^. Dose-dependent anthracycline-induced cardiomyopathy including aberrant arrhythmias, ventricular dysfunction, and heart failure are the most notorious and well-studied cardiovascular toxicities. This toxicity was first described in 1971 in 67 patients treated with Doxorubicin (Dox) for a variety of tumors^1–3^. Despite extensive studies during the past half*-*century, the molecular signaling pathways that underlie the cardiotoxic effects of Dox remain obscure. Several theories have been proposed as plausible underlying mechanisms including mitochondrial dysfunction, increased reactive oxygen species production, defects in iron handling, and contractile failure^4–6^. Despite these findings, the signaling pathways and molecular effectors that drive the cellular defects associated with Dox-associated cardiomyopathy remain obscure.

Although the majority of prior research has implicated nuclear and mitochondrial events as an important etiological aspect of Dox cardiomyopathy, recent discoveries in autophagy have highlighted the renewed interest into these mechanisms^4–6^. However, all these studies to define the role of autophagy in the progression and development of Dox-cardiomyopathy resulted in contradictory conclusion^7,8^. In recent years, several studies demonstrated that Dox impairs the completion of the autophagic process and thereby, suppress myocardial autophagic flux in cardiomyocytes^9–12^. On the contrary, several studies also showed an increased level of autophagy contributing Dox-induced detrimental effects and cell death^13–15^. All of these studies together suggest a potential regulatory and functional role of Dox-induced inhibition/activation of autophagy flux directly contributing to impaired mitochondrial dysfunction. Despite these cellular events, a common hallmark of all Dox-induced cardiotoxic effect is an accumulation of dysfunctional mitochondria, generation of reactive oxygen species (ROS), initiation of mitochondrial oxidative stress and mitochondrial injury^4–6^. All these studies demonstrate that the pathogenesis of Dox-associated cardiomyopathy is a multifocal disease process whose pathological sequelae remains unknown.

In the present study, we aim to define the time point of the onset of impairment in autophagy, mitochondrial dynamics and mitochondrial function that ultimately contribute to the Dox-associated cardiomyopathy. We performed a temporal study using both acute as well as chronic Dox-cardiotoxicity models. To determine the onset of autophagy impairment, we used a combination of biochemical experiments and, reporter GFP-LC3 and mRFP-GFP-LC3 transgenic mice to monitor autophagy *in vivo*. We recapitulated the *in vivo* autophagy and mitochondrial dysfunction data *in vitro* using cultured neonatal cardiomyocytes.

## Results

### Accumulation of autophagosomes and autolysosomes in the acute Dox-associated cardiomyopathy

**Figure 1:**
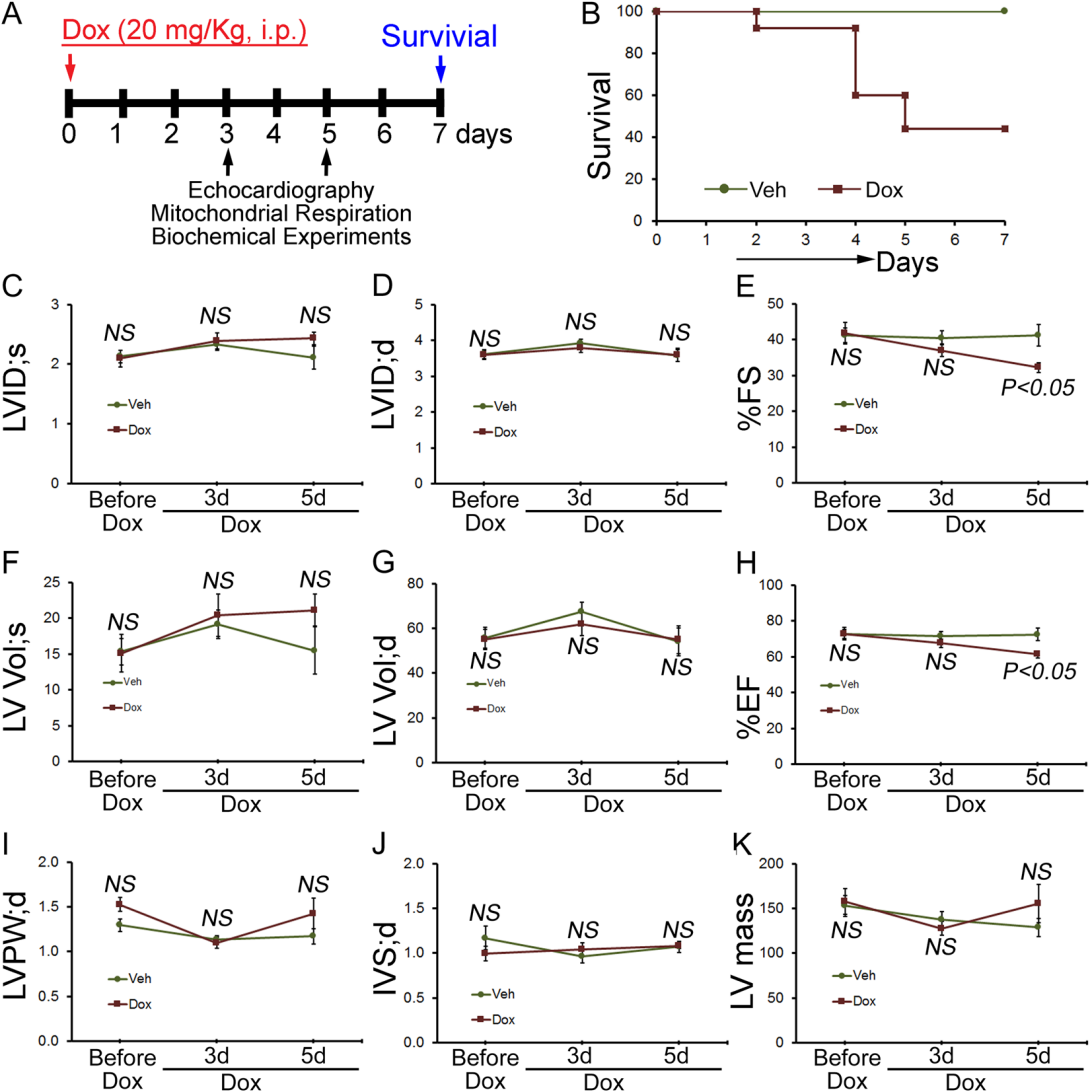
Cardiac function and survival in acute Dox cardiomyopathy mice. (**A**) Schematic of acute Dox administration protocol. FVB/N mice of 8 to 10-weeks of age were treated with a single dose of Dox (20 mg/kg) and vehicle by i.p. injections. (**B**) Kaplan Meier survival curve showing significant mortality in mice after acute Dox (n = 22) treatment compared to Vehicle (n = 10) treated mice. M mode echocardiography was used to examine cardiac function before as well as 3 and 5 days after Dox- and vehicle-injection. (**C**) LV systolic internal dimension (LVID; s). (**D**) LV diastolic internal dimension (LVID; d). (**E**) Percentage fractional shortening (%FS). (**F**) LV systolic volume (LV Vol; s). (**G**) LV diastolic volume (LV Vol; d). (**H**) Percentage ejection fraction (%EF). (**I**) LV diastolic posterior wall thickness (LVPW; d). (**J**) LV diastolic interventricular septum thickness (IVS; d). (**K**) LV mass. Data represent mean ± SEM. *n* = 9 mice for Dox-treatment group and n = 5 mice for vehicle treatment group. *P* value versus vehicle-treated mice by Tukey’s *post hoc* test. NS, not significant.

To study the potential clinical relevance of acute Dox-associated cardiomyopathy, we used the established preclinical model that mimics the response observed clinically by treating the mice with a single injection of Dox (20 mg/kg, i.p)^7,16^. FVB/N mice of 8 to 10-weeks of age comprising both male and female of the same litter were blindly assigned to groups and treated with either a single dose of Dox (20 mg/kg) or vehicle by i.p. injections (Fig. 1A). Acute Dox treatment at this high dose is toxic as the Kaplan Meier survival curves showed significant mortality (56%) in the Dox-treated mice (n = 22) at 7 days compared to vehicle-treated mice (n = 10) (Fig. 1B). The remaining surviving mouse gradually develops cardiac dysfunction as indicated by echocardiographic measurement (Fig. 1C–K). Before Dox-administration, M-mode echocardiographic measurements showed left ventricular function was similar in the randomly allocated vehicle and Dox group mice with similar values for LV internal dimensions in systole and diastole (LVID;s and LVID;d), LV fractional shortening (%FS), volumes in systole and diastole (LV Vol;s and LV Vol;d, respectively), and LV ejection fraction (%EF) (Fig. 1C–K). The Dox-treated mice developed progressive systolic dysfunction, evidenced by decreased %FS and %EF compared with vehicle group (Fig. 1E,H). At three days after the initiation of Dox-treatment, %FS and %EF were markedly reduced in the Dox-treated mice. LV posterior wall thickness (LVPW;d), diastolic thickness of the interventricular septum (IVS;d) and LV mass (Fig. 1I–K) were not changed in the Dox-treated mice. Therefore, acute Dox-associated cardiomyopathy progressively develops systolic cardiac dysfunction.

**Figure 2:**
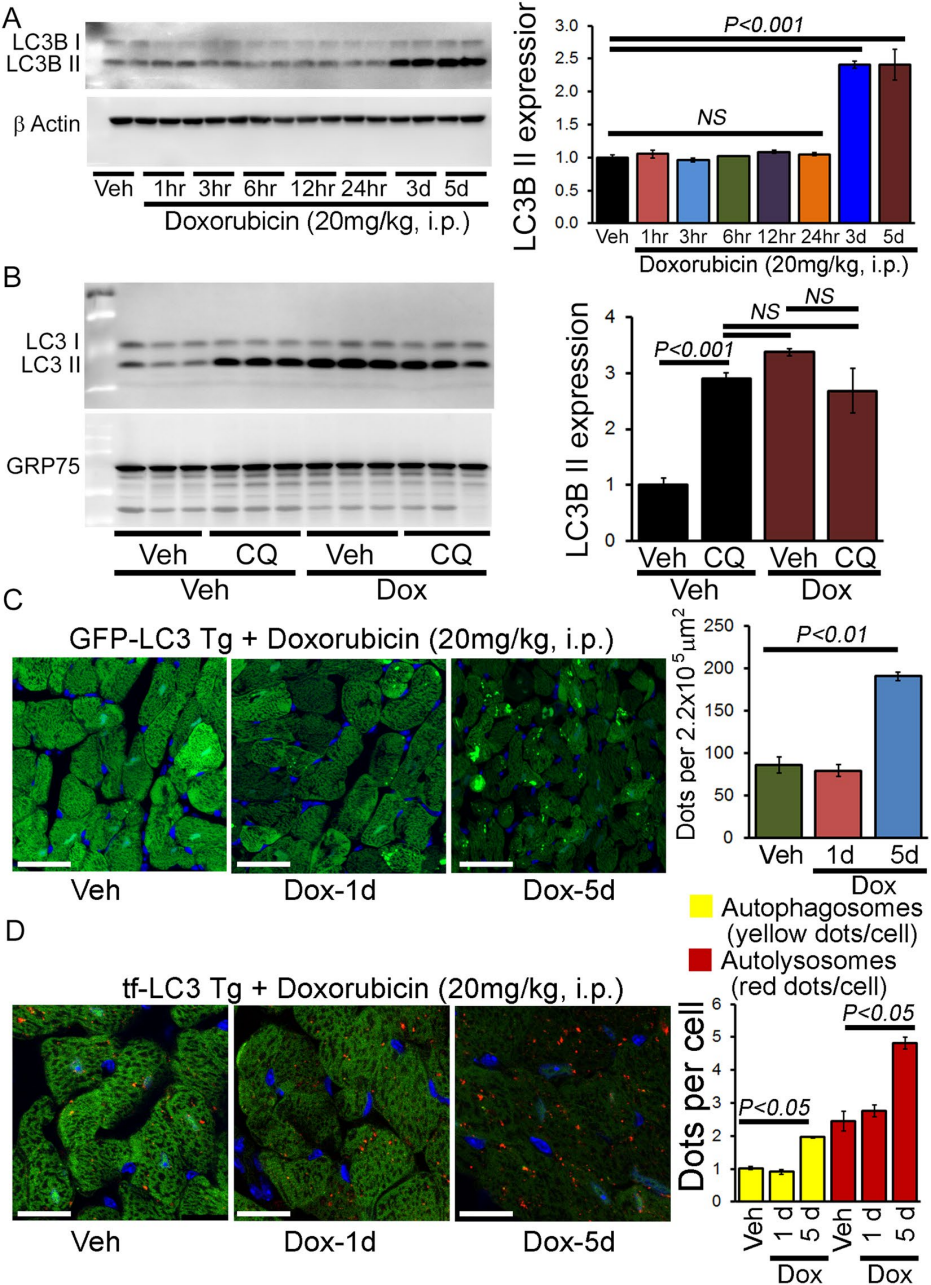
Accumulation of autophagosomes and autolysosomes in the hearts of acute Dox-cardiomyopathy mice. (**A**) Representative Western blot and densitometric quantification showing temporal changes in LC3B II protein levels in the heart after acute Dox (20 mg/kg, i.p.) and vehicle-treatment. β-Actin was used as a loading control. n = 4 mice per group on each time point. (**B**) Autophagic flux assay showed accumulation of autophagosomes resulting from impaired autophagic degradation in acute Dox hearts. GRP75 was used as a loading control. n = 4–6 mice per group. Bars represent mean ± SEM. *P* value versus vehicle-treated mice by Tukey’s *post hoc* test. *NS* = not significant. (**C**) Representative fluorescence images of heart tissue sections from GFP-LC3 Tg mice 1 and 5 days after acute Dox- and vehicle treatment. Quantification of GFP-LC3 puncta/microscopic field in hearts from male GFP-LC3 Tg mouse showing accumulation of autophagosomes in the acute Dox-cardiomyopathy heart at 5 days after Dox-treatment. n = 4–5 hearts per group with 10 microscopic fields (2.2 × 10^5^ μm^2^) per heart section analyzed. Scale bar, 20 μm. (**D**) Representative fluorescence images of heart tissue sections from tf-LC3 Tg mice 1 and 5 days after acute Dox- and vehicle treatment. Quantification of puncta numbers in the heart showing accumulation of autophagosomes (yellow puncta) and autolysosome (red puncta) in the acute Dox-cardiomyopathy heart at 5 days after Dox-treatment. n = 4–5 hearts per group with 10 microscopic fields (2.2 × 10^5^ μm^2^) per heart section analyzed. Scale bar, 20 μm. Bars represent mean ± SEM. *P* value versus vehicle-treated mice by Tukey’s *post hoc* test. *NS* = not significant.

Extensive studies on Dox-cardiomyopathy shows activation/inhibition of autophagy contributes to the progression and development of cardiomyopathy^7,8^. We performed a temporal study to determine the time point associated with the onset of impaired autophagy following acute Dox-treatment to study the potential clinical relevance of autophagy in acute Dox-associated cardiomyopathy^14,17^. Western blot analysis showed time-dependent accumulation of LC3B II at both 3 and 5 days post injection (Fig. 2A). LC3B II is present on isolation membranes, autophagosomes, and much less on autolysosomes^18^. Therefore, the increase in LC3B II indicates the accumulation of autophagosomes and autolysosomes resulting from either activation of autophagosome synthesis or blockade of a downstream autophagic degradation process. To distinguish between these two possibilities, we administered either chloroquine (CQ) (50 mg/kg, i.p. once daily for 5 days) or saline to inhibit lysosomal degradation and determine LC3B II levels by Western blot analysis in the Dox-treated animals^19,20^. Treatment of control animals with CQ resulted in a significant increase in LC3B II levels, reflecting cardiac autophagic flux under basal conditions. However, Dox-treated animals manifested no increase in LC3B II levels with CQ treatment suggesting that autophagosomes/autolysosomes accumulation occurred due to inhibition of autophagic degradation (Fig. 2B).

Next, we used cardiomyocyte-specific GFP-LC3 transgenic (Tg) autophagy reporter mouse to monitor Dox-induced autophagosomes accumulation *in vivo* selectively^19,21^. During the process of autophagy, autophagosomes undergo a maturation process consisting of multiple fusions with endosomes and lysosomes, which provide an acidic environment and digestive function to the interior of the autophagosome. The GFP-LC3 is sensitive to acidic pH and ceases to fluoresce once autophagosomes fuse with the lysosome, resulting in the inability to detect autolysosomes^22^. Acute Dox-treatment showed accumulation of GFP-labelled green puncta indicating autophagosomes accumulation at 5 days after Dox-administration (Fig. 2C).

**Figure 3:**
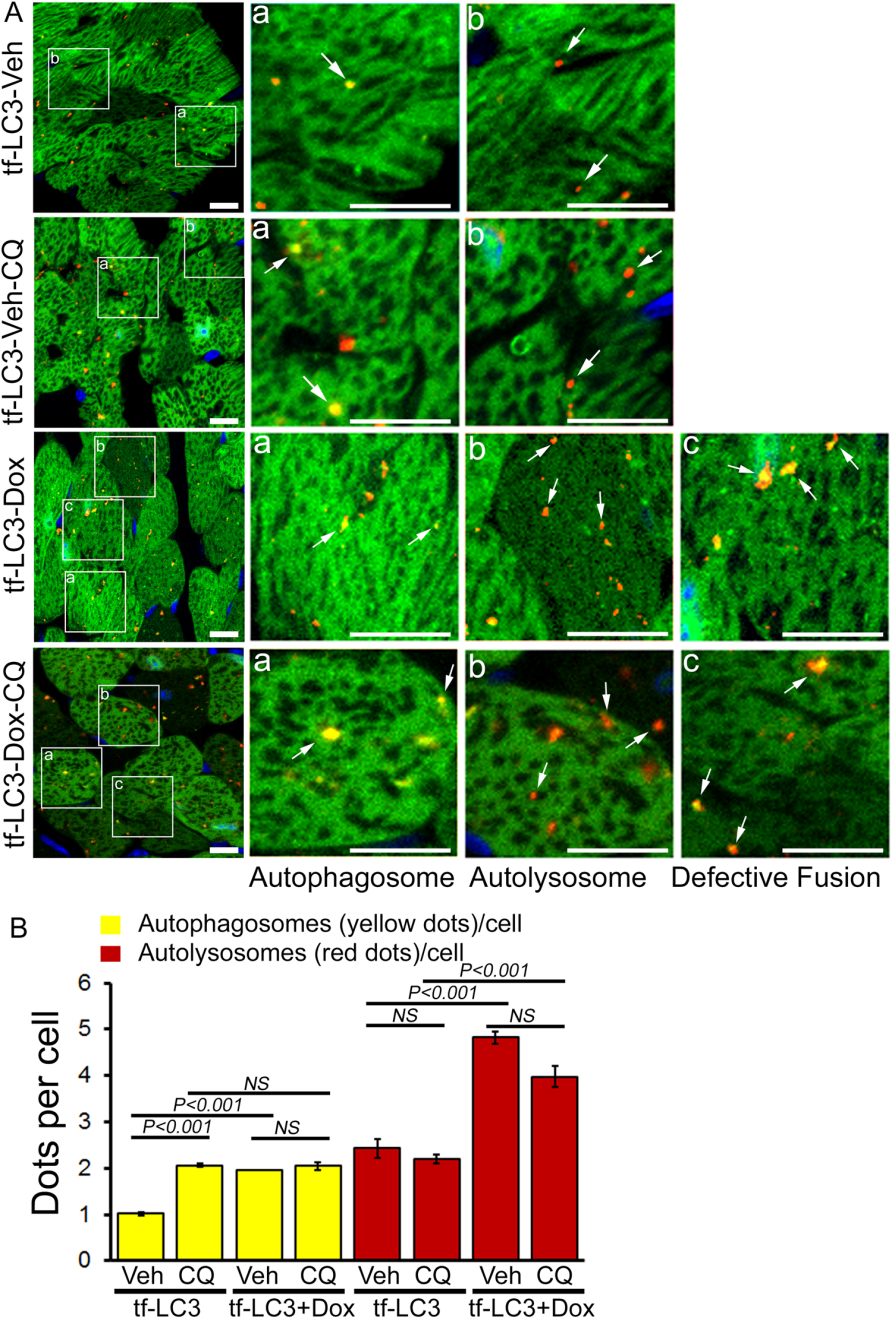
Inhibition of autophagic degradation process in the hearts of acute Dox-cardiomyopathy mice. (**A**) Representative fluorescence images of heart tissue sections from tf-LC3 Tg mice 5 days after acute Dox as well as saline administered heart treated with chloroquine (CQ) or vehicle. Autophagic flux assay showed an accumulation of autophagosomes (yellow puncta), autolysosomes (red puncta) and defective autophagosome/autolysosome fusion (red and yellow pucta coming together) in acute Dox-treated hearts (indicated by white arrow). (**B**) Quantification of puncta numbers in the heart showing accumulation of autophagosomes and autolysosome in the acute Dox-cardiomyopathy heart at 5 days after Dox-treatment with or without CQ treatment. n = 4–5 hearts per group with 10 microscopic fields (2.2 × 10^5^ μm^2^) per heart section analyzed. Scale bar, 10 μm. Bars represent mean ± SEM. *P* value versus vehicle-treated mice by Tukey’s *post hoc* test. *NS* = not significant.

Next, we used the tandem fluorescent mRFP-GFP-LC3 (tf-LC3) Tg reporter mice to quantitate the Dox-induced relative changes in autophagosomes versus autolysosomes accumulation^19,23^. These tf-LC3 Tg mice can monitor both autophagosome and autolysosome formation simultaneously, as LC3 puncta labeled with both GFP and mRFP (yellow puncta) represent autophagosomes. In the lysosome, the fluorescence of GFP is quenched due to its low pH whereas that of mRFP (red puncta) is stable representing autolysosomes. Formation of autophagosomes causes an increase in the number of GFP-positive/mRFP-positive (yellow) puncta, and the puncta become GFP-negative/mRFP-positive (red) upon fusion with lysosomes. Autophagy induction or inhibition of autophagic degradation results in the increase in both yellow and red puncta^19,23^. In acute Dox-treated tf-LC3 Tg mice, we observed a significant accumulation of autophagosomes (yellow puncta, LC3 puncta labeled with both GFP and mRFP) and autolysosomes (red puncta, mRFP labeled) (Fig. 2D). Next, we treated a group of Dox and vehicle-treated mice with CQ (50 mg/kg, i.p.) to confirm the accumulation of autophagosomes and autolysosomes resulting from the inhibition of autophagic degradation process. The yellow and red puncta counts with or without Dox-treatment at 5 days (as in Fig. 2D) were used as a control group for the autophagy flux data in Fig. 3. Treatment of tf-LC3 Tg animals with CQ resulted in a significant increase in yellow puncta and a slight decrease in red puncta counts, reflecting cardiac autophagic flux under basal conditions (Fig. 3A,B). However, CQ treatment to Dox-administered tf-LC3 Tg mice manifested no increase in yellow/red puncta counts suggesting that autophagosomes and autophagosomes accumulation occurred due to inhibition of autophagic degradation process (Fig. 3A,B).

Interestingly, some of the yellow puncta in Dox-treated tf-LC3 mice showed distinct characteristics compared to the saline-treated mice^24^. In the Dox-treated hearts, the yellow puncta appear in the middle or adjacent to the red puncta suggesting either defects in autophagosome/lysosome fusion or inability to the quenching of the GFP signal in autolysosome which is sensitive to the pH of the lysosome (Fig. 3A, indicated by white arrow). Though CQ treatment is known to induce lysosomal pH inhibiting autophagosomes fusion with lysosomes, the CQ-treated tf-LC3 Tg mice (without Dox) appears as the clear yellow puncta. All these autophagy flux data suggest that Dox-treatment impairs autophagy flux resulting in the accumulation of autophagosomes and autolysosomes as well as defective autophagosome/autolysosome fusion.

### Changes in mitochondrial dynamics and OXPHOS regulatory protein expression in acute Dox-associated cardiomyopathy

**Figure 4:**
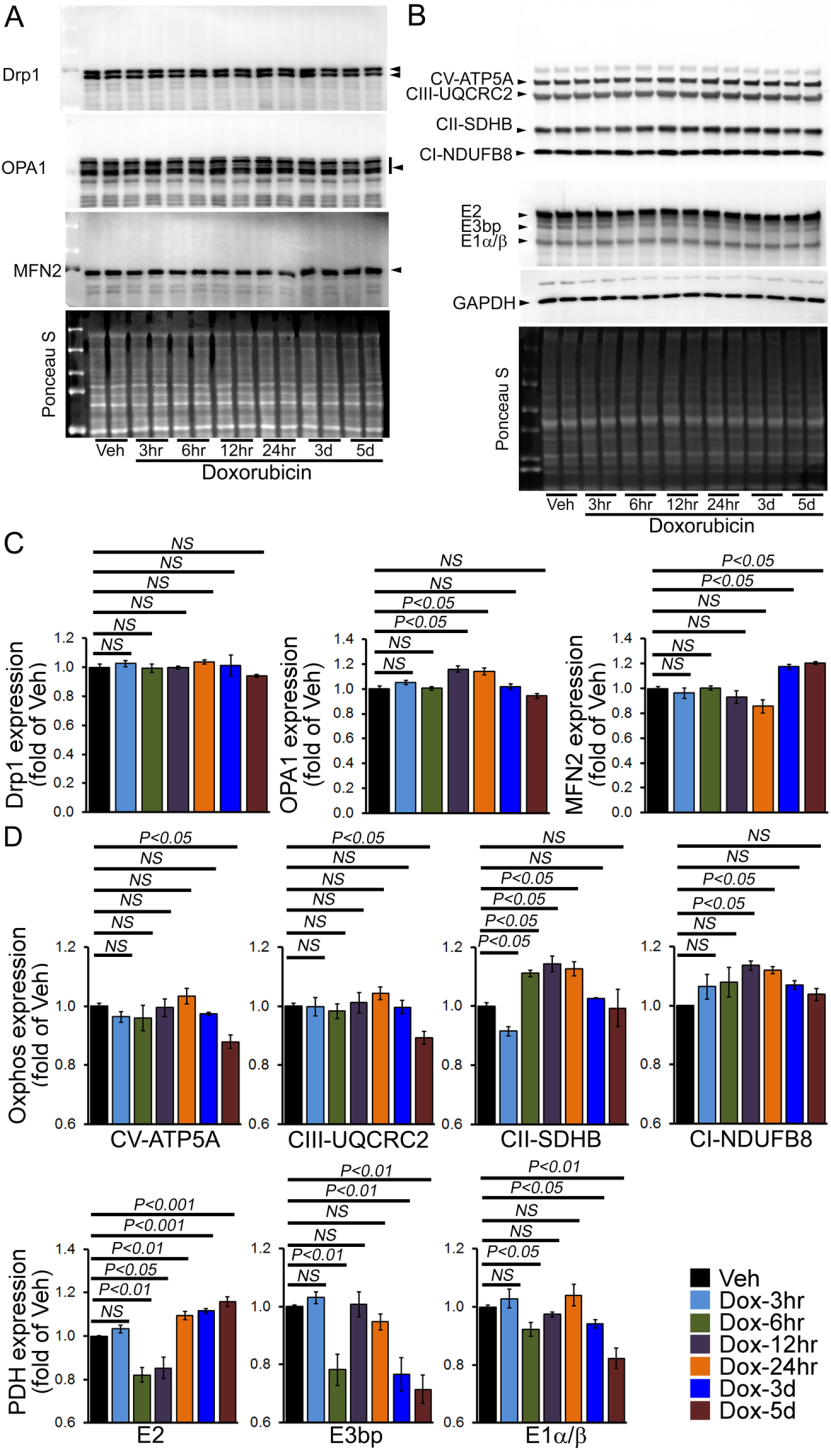
Expression of mitochondrial dynamics and OXPHOS regulatory protein in the hearts of acute Dox-cardiomyopathy mice. (**A**) Representative Western blot of the whole cell fraction showing expression of mitochondrial dynamic regulatory proteins in the acute Dox-treated hearts: Drp1, OPA1, and MFN2. Ponceau S protein staining of the transfer membrane confirmed approximately equal loading across the gel. (**B**) Representative Western blot showing expression of Complex I, Complex II, Complex III, Complex V and PDH complex protein derived from the whole cell fraction isolated from acute Dox-treated hearts at different time points. Ponceau S protein stain of the transfer membrane was used to confirm approximately equal loading. (**C**) Densitometric quantification of the temporal changes in Drp1, OPA1, and MFN2 protein expression in Dox-treated hearts. (**D**) Densitometric quantification of OXPHOS complex and PDH complex protein. Bars represent mean ± SEM. n = 4 mice per group at each time point. *P* values were determined by Tukey’s *post-hoc* test. *NS* = not significant.

Along with impaired autophagy, Dox-cardiotoxicity has been shown to be associated with accumulation of dysfunctional mitochondria which may ultimately result in impaired mitochondrial dynamics. Therefore, we investigated the effect of Dox-treatment on mitochondrial fission (i.e., DRP1) and fusion proteins (i.e., OPA1, and Mfn2). Immunoblot analysis showed no significant changes in DRP1 protein expression in whole cell lysates from Dox-treated hearts compared with the vehicle group (Fig. 4A,C). We observed significantly increased expression of OPA1 12 h and 24 h post-Dox-treatment. MFN2 levels were also significantly increased 3 d and 5 d following Dox-treatment compared with the vehicle group (Fig. 4A,C). Ponceau-S staining of the Western blots was used to confirm equivalent loading. During mitochondrial fission, Drp1 translocate from the cytosol to prospective fission sites on the mitochondrial surface. Western blot and densitometric quantification showed a similar level of Drp1, OPA1 and MFN2 expression in the mitochondrial fraction of Dox-treated hearts at 5 days compared to vehicle-treated hearts (Fig. S1A,B). The purity of the mitochondrial fractionation confirmed by the absence of GAPDH in the mitochondrial fraction, and we used GAPDH protein expression in the cytosolic fraction as a positive control. Western blots for COXIV were used to confirm the mitochondrial extracts using the same membrane. Ponceau S protein staining of the transfer membrane were used to confirm approximately equal loading and transfer across the gel. Therefore, acute Dox-treatment has minimal effects on the expression of mitochondrial fusion regulatory proteins in the mitochondrial fraction suggesting minimal effect in mitochondrial dynamics.

Next, we examined OXPHOS protein levels in whole cell lysates prepared from Dox- and vehicle-treated mice hearts. Dox hearts showed significantly decreased levels of Complex V and III in the whole cell lysates at 5d. In contrast, Complex II and Complex I expression was significantly increased at 12 and 24 h post-Dox-treatment (Fig. 4B,D). Expression of the E2 protein of the pyruvate dehydrogenase (PDH) complex was significantly increased starting from the 24 h following Dox-treatment. In contrast, the expression of E3bp and E1α/β protein levels were significantly decreased in the whole cell lysate of the Dox-treated hearts at 3d and 5d after Dox-treatment. Ponceau S staining of proteins was used to confirm equal loading (Fig. 4B,D). Collectively, we found that the acute Dox-treated hearts showed altered expression of mitochondrial oxidative phosphorylation regulatory protein.

### Suppression of mitochondrial respiration in acute Dox-associated cardiomyopathy

**Figure 5:**
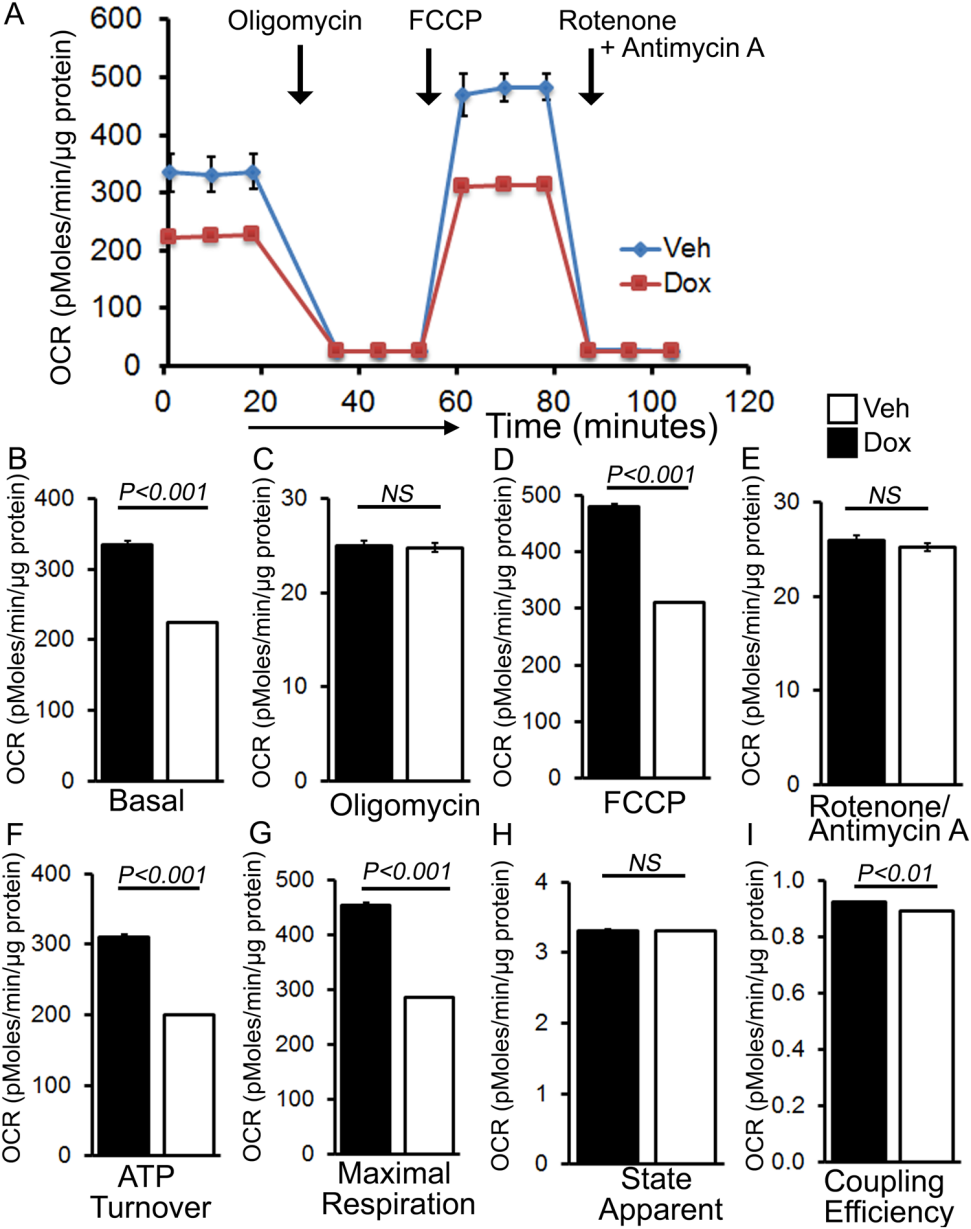
Suppression of mitochondrial respiration in the hearts of acute Dox-cardiomyopathy mice. (**A**) Mitochondrial oxygen consumption rate (OCR) profiles in isolated mitochondria from 3 days after acute Dox-treated hearts. Arrow indicates the sequential addition of oligomycin (1 µM), FCCP (4 µM), and rotenone (0.5 µM) plus antimycin A (0.5 µM). OCR profile is expressed as pMolesO_2_/min/µg of protein. Graph showing OCR under (**B**) baseline as well as with the addition of (**C**) oligomycin, (**D**) FCCP, and (**E**) rotenone plus antimycin A. Key parameters of mitochondrial function, including (**F**) ATP turnover, (**G**) maximal respiration, (**H**) state apparent and (**I**) coupling efficiency were significantly decreased in Dox mice. Bars represent mean ± SEM. n = 4–6 mice per group. *P* values were determined by Tukey’s *post-hoc* test.

To ascertain if the mitochondria in the Dox-treated hearts shows defective mitochondrial bioenergetics, we isolated mitochondria from both Dox- and vehicle-treated mouse hearts at 3 days after treatment and measured mitochondrial respiration. Real-time oxygen consumption rates (OCRs) in isolated mitochondria show that basal respiration, representing the sum of all physiological mitochondrial oxygen consumption, was decreased in the mitochondria from Dox-treated heart, indicating lower respiratory function compared with vehicle hearts (Fig. 5A,B). The injection of oligomycin, an ATP synthase inhibitor, leads to a decrease in basal respiration that is reflective of oxygen consumption used to generate ATP (Fig. 5C). The addition of carbonyl cyanide-p-trifluoromethoxy-phenylhydrazone (FCCP) uncouples respiration from oxidative phosphorylation and allows for the measurement of maximal OCR, which was lower in Dox-heart mitochondria (Fig. 5D), indicating lower overall mitochondrial activity. The extent of non-mitochondrial oxygen-consuming processes was estimated by inhibiting the respiratory chain with rotenone and antimycin A; there was no significant change in Dox-heart mitochondria (Fig. 5E). The ATP turnover measured by ATP-linked respiration subtracted from the basal OCR, was significantly decreased in Dox mitochondria (Fig. 5F). The maximum respiration calculated by non-mitochondrial respiration subtracted from FCCP-stimulated OCR was also significantly lower in Dox-heart mitochondria (Fig. 5G). State_apparent_ was calculated to determine the apparent respiratory rate that provides an estimate of the relative mitochondrial work being used by the cells under basal conditions. State_apparent_ was calculated as follows: State_apparent_ = 4-(Basal OCR-Oligomycin OCR)/(FCCP OCR–Oligomycin OCR)^25,26^. Mitochondria isolated from Dox-hearts showed similar level of State_apparent_ (Fig. 5H)^26^. Coupling efficiency was calculated as the fraction of basal mitochondrial OCR used for ATP synthesis (ATP-linked OCR/basal OCR)^25,27^. Mitochondria isolated from Dox-hearts also showed lower coupling efficiency indicating a lower proportion of oxygen consumed to drive ATP synthesis compared with that driving proton leak (Fig. 5I).

Similarly, mitochondria isolated at 5 days after Dox-and vehicle treatment also showed similar changes in mitochondrial respiration (Supplement Fig. S2A–I). Interestingly, mitochondria isolated at 5 days after Dox-treatment showed lower State_apparent_ indicating a decrease in overall flux through the respiratory chain (Supplement Fig. S2H)^26^. Therefore, mitochondria isolated from hearts at 3d and 5d after Dox-treatment are functionally compromised.

### Accumulation of autophagosomes and autolysosomes in chronic Dox-associated cardiomyopathy

**Figure 6:**
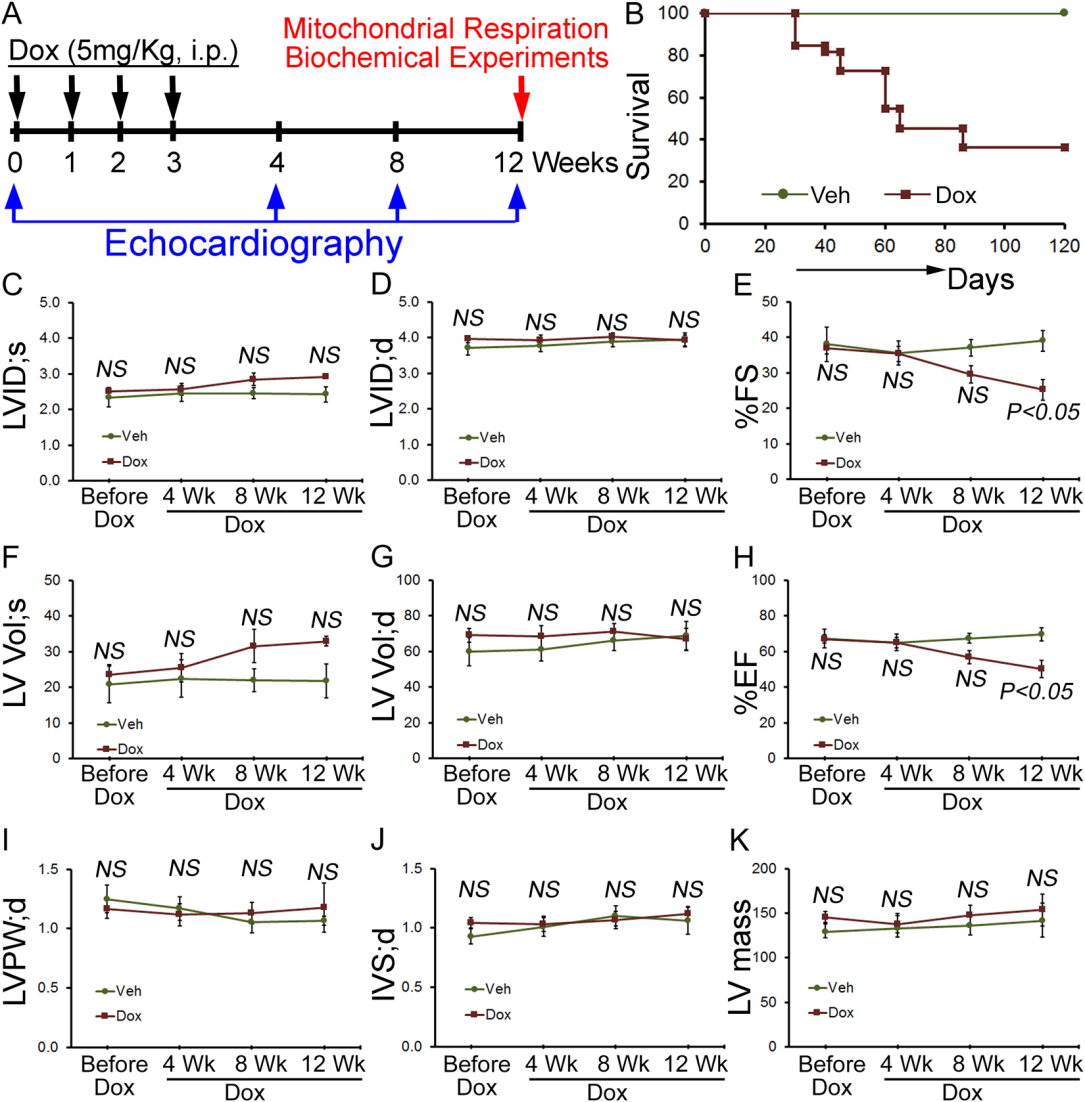
Cardiac function and survival in chronic Dox-associated cardiomyopathy mice. (**A**) Schematic of chronic Dox administration protocol. FVB/N mice of 8 to 10-weeks of age were subjected to four serial Dox (5 mg/kg) injections weekly by i.p. (**B**) Kaplan Meier survival curve showing mortality in mice after chronic Dox (n = 13) treatment compared to Vehicle (n = 10) treated mice. M mode echocardiography was used to examine cardiac functions before as well as 4, 8 and 12 weeks after Dox and vehicle-injection. (**C**) LV systolic internal dimension (LVID; s). (**D**) LV diastolic internal dimension (LVID; d). (**E**) Percentage fractional shortening (%FS). (**F**) LV systolic volume (LV Vol; s). (**G**) LV diastolic volume (LV Vol; d). (**H**) Percentage ejection fraction (%EF). (**I**) LV diastolic posterior wall thickness (LVPW; d). (**J**) LV diastolic interventricular septum thickness (IVS; d). (**K**) LV mass. *n* = 15 mice for Dox-treatment group and n = 5 mice for vehicle treatment group. Bars represent mean ± SEM. *P* value versus vehicle-treated mice by Tukey’s *post hoc* test. NS, not significant.

To induce the chronic Dox-associated cardiomyopathy, we treated mice with 4 once-per-week low-dose doxorubicin injections (5 mg/kg; i.p.) (Fig. 6A). This dose was selected because the pharmacokinetics of plasma doxorubicin after 1 dose of 5 to 6 mg/kg in mice is comparable to that seen in patients after a standard dose of Dox-treatment (60 mg/m^2^)^12,28–30^. FVB/N mice of 8 to 10-weeks of age comprising both male and female of the same litter were randomly assigned and treated with Dox- and vehicle treatments (Fig. 6A). A Kaplan Meier survival curve showed significant mortality (64%) in Dox-treated mice (n = 13) at 12 weeks compared to vehicle-treated mice (n = 10) (Fig. 6B). Before Dox-administration, M-mode echocardiographic measurements showed left ventricular systolic function was similar in these mice (Fig. 6C–K). The Dox-treated mice developed progressive systolic dysfunction at 8–12 weeks after the initiation of Dox-treatment, evidenced by decreased %FS and %EF compared with vehicle group (Fig. 6E,H). LV posterior wall thickness (LVPW;d), the diastolic thickness of the interventricular septum (IVS;d) and LV mass (Fig. 6I–K) were not changed in the chronic Dox-treated mice. Therefore, chronic Dox-associated cardiomyopathy progressively develops systolic cardiac dysfunction.

**Figure 7:**
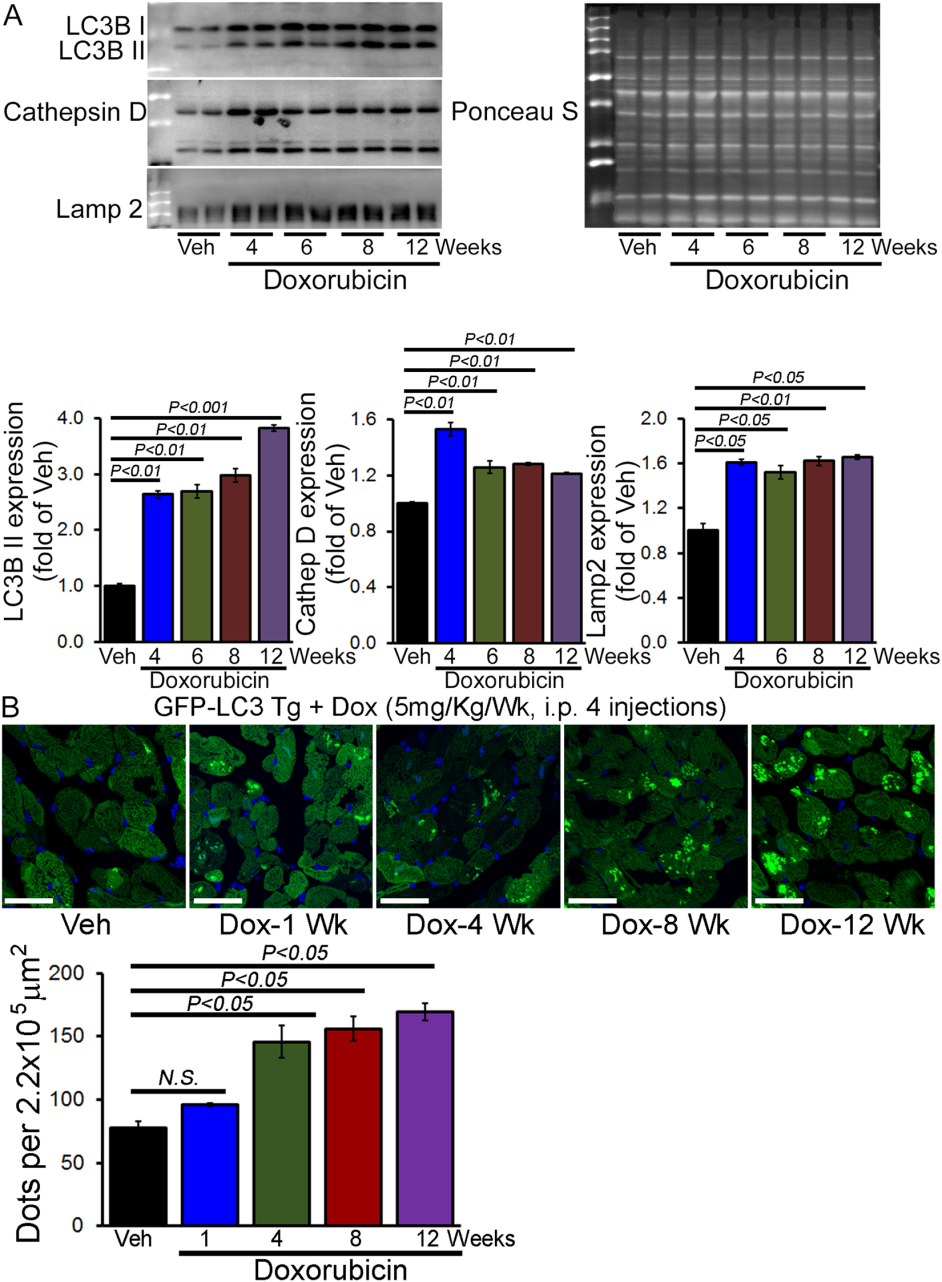
Accumulation of autophagosomes and autolysosomes in the hearts of chronic Dox-cardiomyopathy mice. (**A**) Representative Western blot and densitometric quantification showing temporal changes in LC3B, Cathepsin D and Lamp 2a protein levels in the heart of 4, 6, 8 and 12 weeks after Dox- and vehicle-treatment. Ponceau S protein stain of the transfer membrane was used to confirm approximately equal loading. n = 4 hearts per group on each time point. Bars represent mean ± SEM. (**B**) Representative fluorescence images of heart tissue sections from GFP-LC3 Tg mice 1, 4, 8, and 12 weeks after Dox- and vehicle treatment. Quantification of GFP-LC3 puncta/microscopic field in hearts from male GFP-LC3 Tg mouse showing accumulation of autophagosomes in the chronic Dox-cardiomyopathy heart after Dox-treatment. n = 4–5 hearts per group with 10 microscopic fields (2.2 × 10^5^ μm^2^) per heart section analyzed. Scale bar, 20 μm. Bars represent mean ± SEM. *P* value versus vehicle-treated mice by Tukey’s *post hoc* test. *NS* = not significant.

Similar to acute Dox-effects, Western blot analysis showed time-dependent accumulation of autophagosomes as indicated by LC3B II level starting at 4 weeks following Dox-injection (Fig. 7A); mice still have normal heart function at the time point. We also found time-dependent accumulation of lysosomal protein Lamp2a and Cathepsin D starting from 4 weeks after Dox-treatment (Fig. 7A). We confirmed the accumulation of autophagosomes *in vivo* by using the GFP-LC3 Tg mice with chronic Dox- and vehicle treatment. Quantification of GFP-labelled green puncta also confirmed time-dependent autophagosomes accumulation starting from 4 weeks after Dox-treatment, indicating inhibition of the autophagic degradation process (Fig. 7B).

### Changes in mitochondrial dynamics and OXPHOS regulatory protein expression in chronic Dox-associated cardiomyopathy

**Figure 8:**
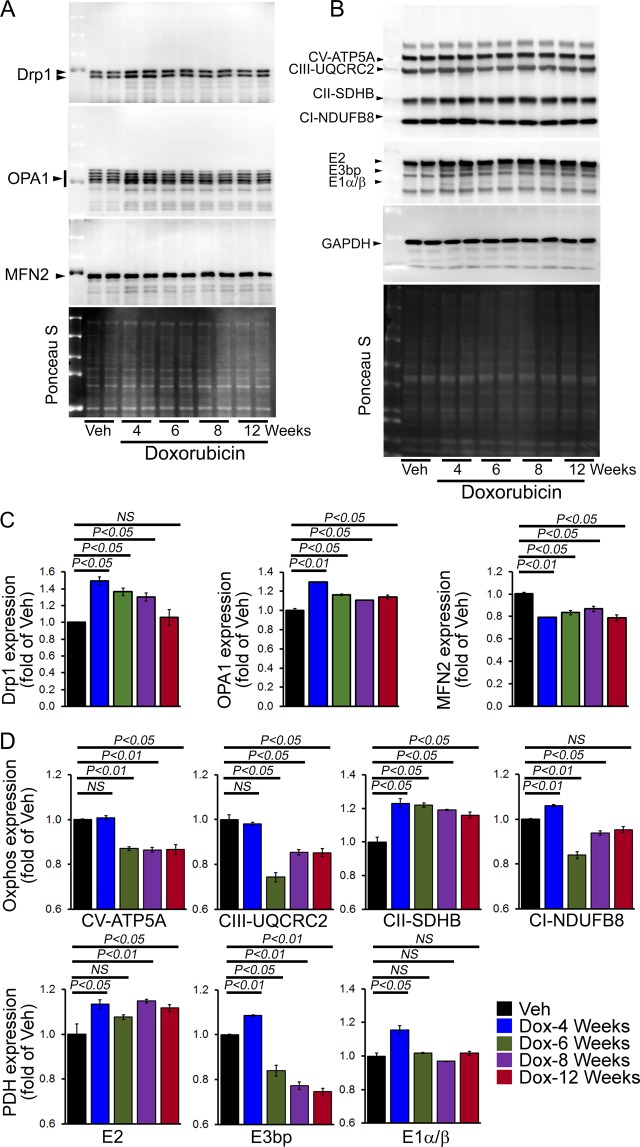
Altered expression of mitochondrial dynamics and OXPHOS regulatory protein in the hearts of chronic Dox-cardiomyopathy mice. (**A**) Representative Western blot of the whole cell fraction showing expression of mitochondrial dynamic regulatory proteins in the acute Dox-treated hearts: Drp1, OPA1, and MFN2. Ponceau S protein staining of the transfer membrane confirmed approximately equal loading across the gel. (**B**) Representative Western blot showing expression of Complex I, Complex II, Complex III, Complex V and PDH complex protein derived from the whole cell fraction isolated from acute Dox-treated hearts at different time points. Ponceau S protein stain of the transfer membrane was used to confirm approximately equal loading. (**C**) Densitometric quantification of the temporal changes in Drp1, OPA1, and MFN2 protein expression in Dox-treated hearts. (**D**) Densitometric quantification of OXPHOS complex and PDH complex protein. Bars represent mean ± SEM. n = 4 mice per group at each time point. *P* values were determined by Tukey’s *post-hoc* test. *NS* = not significant.

Next, we investigated the effect of Dox-treatment on mitochondrial fission (i.e., DRP1) and fusion protein (i.e., OPA1, and Mfn2) expression. Immunoblot analysis showed significantly increased levels DRP1 protein expression in whole cell lysates from Dox-treated hearts compared with vehicle group starting at the first week of Dox-treatment (Fig. 8A,C). We observed significantly increased expression of OPA1 starting from 4 weeks after Dox-treatment. In contrast, MFN2 expression levels were also significantly decreased starting after 1 week of Dox-treatment compared with the vehicle group (Fig. 8A,C). Ponceau-S staining of the Western blots was used to confirm equivalent loading. We also quantitated the level of Drp1, OPA1 and MFN2 expression in the mitochondrial fraction of Dox-treated hearts at 12 weeks after Dox and vehicle-treated hearts (Fig. S3A,B). Similar to the whole cell lysate, the Drp1 expression remains similar and OPA1 expression level significantly increased in the mitochondrial fraction. In contrast to the whole cell lysate, MFN2 expression significantly increased in the mitochondrial fraction (Fig. S3A,B). GAPDH protein expression in the cytosolic fraction was used as a positive control and the purity of the mitochondrial fractionation was confirmed by the absence of GAPDH in the mitochondrial fraction purity. Western blots for COXIV were used to confirm the mitochondrial extracts using the same membrane. Ponceau S protein staining of the transfer membrane confirmed approximately equal loading and transfer across the gel. Therefore, chronic Dox-treatment causes significant alterations in the mitochondrial dynamics of protein expression.

Because the Dox-treated mouse hearts showed defective mitochondrial dynamics, we compared OXPHOS protein levels in whole cell lysates prepared from Dox- and vehicle-treated mouse hearts of mixed gender. Western blot analysis showed significantly decreased levels of Complex V and III in the whole cell lysates starting at 6–12 weeks after Dox-treatment (Fig. 8B,D). In contrast, Complex II and Complex I protein expression was significantly increased at 4 weeks after Dox-treatment (Fig. 8B,D). Expression of the E2 protein of the PDH complex was significantly increased beginning at 4 weeks after Dox-treatment. In contrast, the expression of E3bp and E1α/β protein level was significantly decreased in the whole cell lysate of the Dox-treated hearts (Fig. 8B,D). Ponceau S staining of proteins was used to confirm equal loading. Collectively, we found that the chronic Dox-treated hearts showed altered mitochondrial dynamics and oxidative phosphorylation regulatory protein expression starting before the onset of cardiac functional decline.

### Suppression of mitochondrial respiration in chronic Dox-associated cardiomyopathy

**Figure 9:**
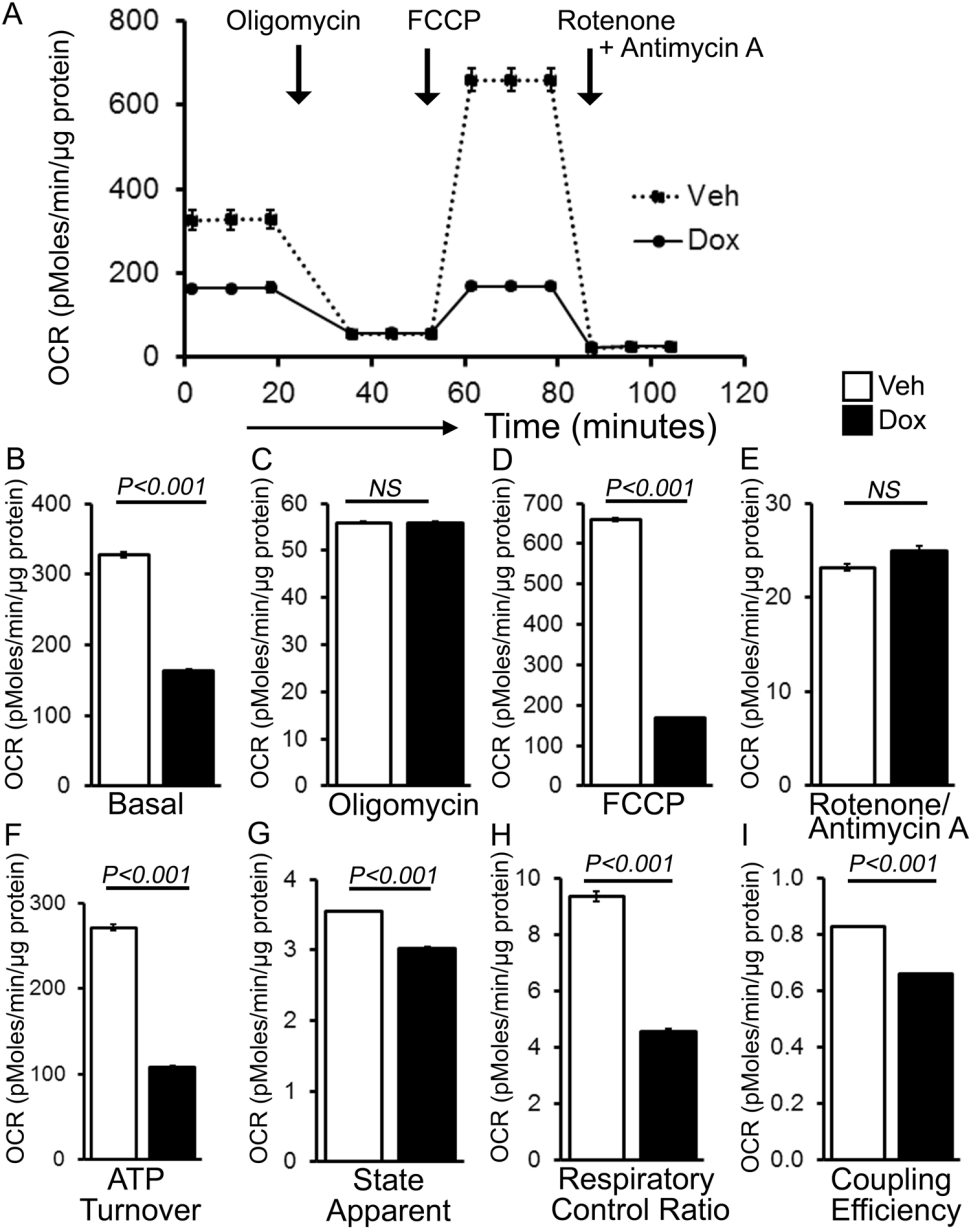
Suppression of mitochondrial respiration in the hearts of chronic Dox-cardiomyopathy mice. (**A**) Mitochondrial oxygen consumption rate (OCR) profiles in isolated mitochondria from 12 weeks after Dox- and vehicle-treated hearts. Arrow indicates the sequential addition of oligomycin (1 µM), FCCP (4 µM), and rotenone (0.5 µM) plus antimycin A (0.5 µM). OCR profile are expressed as pMolesO_2_/min/µg of protein. Graph showing OCR under (**B**) baseline as well as with the addition of (**C**) oligomycin, (**D**) FCCP, and (**E**) rotenone plus antimycin A. Key parameters of mitochondrial function, including (**F**) ATP turnover, (**G**) state apparent, (**H**) respiratory control ratio and, (**I**) coupling efficiency were significantly decreased in Dox mice. Bars represent mean ± SEM. n = 6 mice per group. *P* values were determined by Tukey**’**s *post-hoc* test.

The chronic Dox-treated hearts showed altered expression of oxidative phosphorylation regulatory proteins, so next, we isolated the mitochondrial 12 weeks after the Dox-treatment and evaluated mitochondrial respiratory profile using the XF Extracellular Flux Analyzer (Seahorse Bioscience). Real-time OCRs in isolated mitochondria show that basal respiration, representing the sum of all physiological mitochondrial oxygen consumption, was decreased in the Dox-hearts, indicating lower respiratory function compared with vehicle hearts (Fig. 9A,B). In addition to low basal respiration, Dox-heart mitochondria did not show any difference in ATP-linked OCR (Fig. 9C). The measurement of maximal OCR after addition of FCCP was lower in Dox mitochondria (Fig. 9D), indicating lower overall mitochondrial activity. The extent of non-mitochondrial oxygen-consuming processes was not significantly different in Dox-heart mitochondria (Fig. 9E). The ATP turnover measured by ATP-linked respiration subtracted from the basal OCR, was significantly decreased in Dox mitochondria (Fig. 9F). Mitochondria isolated from Dox-hearts also showed lower State_apparent_ indicating a decrease in overall flux through the respiratory chain (Fig. 9G)^26^. Respiratory control ratio (RCR) was calculated as (maximal OCR/oligomycin-insensitive OCR) which indicates the tightness of the coupling between respiration and oxidative phosphorylation. RCR value is sensitive to changes in substrate oxidation and proton leak, but not ATP turnover^27^. RCR was significantly lower in the mitochondria from Dox-hearts indicating a lower potential for substrate oxidation and ATP turnover. Dox-hearts also showed lower coupling efficiency indicating lower proportion of the oxygen consumed to drive ATP synthesis compared with that driving proton leak (Fig. 9I). We conclude that mitochondria isolated from chronic Dox cardiomyopathy mice hearts are functionally compromised.

### Inhibition of autophagic flux and suppression of mitochondrial respiration by Dox-treatment in cultured cardiomyocytes

**Figure 10:**
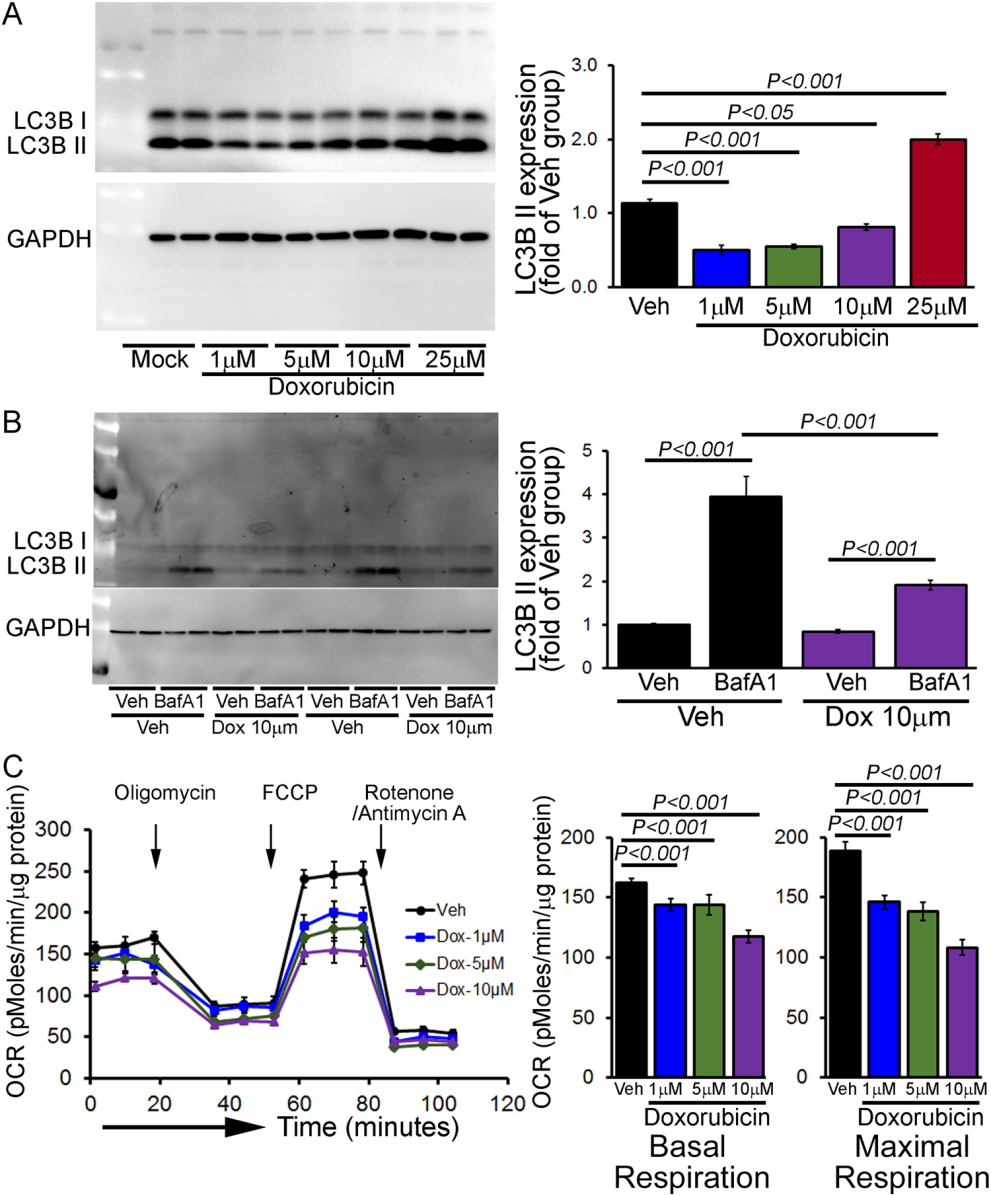
Inhibition of autophagic flux and suppression of mitochondrial respiration by Dox-treatment in cultured cardiomyocytes. (**A**) Representative Western blot and densitometric quantification showing LC3B II expression at different doses of Dox (1–25 μM, 24 hours) in NRCs. GAPDH was used as a loading control. n = 6 independent experiments. (**B**) Inhibition of autophagic flux by 10 μM Dox (24 hours) in NRC examined by immunoblotting of LC3B II. Bafilomycin A1 (50 μg/mL) was added for 4 hours to block lysosomal degradation, and GAPDH was used as a loading control. n = 6 independent experiments. Bars represent mean ± SEM. (**C**) Dox treatment (1–10 μM Dox, 24 hours) dose-dependently suppress mitochondrial oxygen consumption rate (OCR) profiles in NRCs. Arrow indicates the sequential addition of oligomycin (1 µM), FCCP (4 µM), and rotenone (0.5 µM) plus antimycin A (0.5 µM). OCR profile are expressed as pMolesO_2_/min/µg of protein. Graph showing OCR under baseline as well as with the addition of oligomycin, FCCP, and rotenone plus antimycin A. Key parameters of the mitochondrial function, including basal and maximal respiration were dose-dependently decreased in Dox-treated NRCs. Bars represent mean ± SEM. n = 5 wells per group. *P* values were determined by Tukey’s *post-hoc* test.

To determine if Dox-treatment directly results in altered autophagy and mitochondrial respiratory dysfunction, we treated neonatal rat cardiomyocytes (NRCs) with Dox- and vehicle. We then performed Western blot quantification to observe the LC3B II expression level at different doses of Dox (1, 5, 10 and 25 μM, 24 hours) in NRCs. In contrast to the *in vivo* Dox-effects, lower doses of Dox showed decreased levels of LC3B II expression but higher doses of Dox (25 μM) showed increased LC3B II accumulation (Fig. 10A). To confirm that this decreased level of LC3B II was due to impaired autophagy, we performed an autophagic flux assay by using 10 μM Dox (24 hours) treatment in NRC. Bafilomycin A1 (50 nmol/L) was added for 4 hours to block lysosomal degradation, and GAPDH was used as a loading control (Fig. 10B). The autophagy flux assay showed inhibition of autophagic activity in Dox-treated NRCs.

To ensure that the decreased OCR we observed in isolated mitochondria from acute and chronic Dox-treated mouse hearts was not an artifact due to the mitochondrial isolation process, we measured OCR in intact NRCs treated with either Dox or vehicle (Fig. 10C). NRCs exposed to Dox (1, 5 and 10 μM, 24 hours) showed a dose-dependently decreased in OCR at baseline as well as with the addition of oligomycin (1 µM), FCCP (4 µM), and rotenone (0.5 µM) plus antimycin A (0.5 µM) (Fig. 10C). Key parameters of the mitochondrial function, including basal and maximal respiration, were dose-dependently decreased in Dox-treated NRCs.

**Figure 11:**
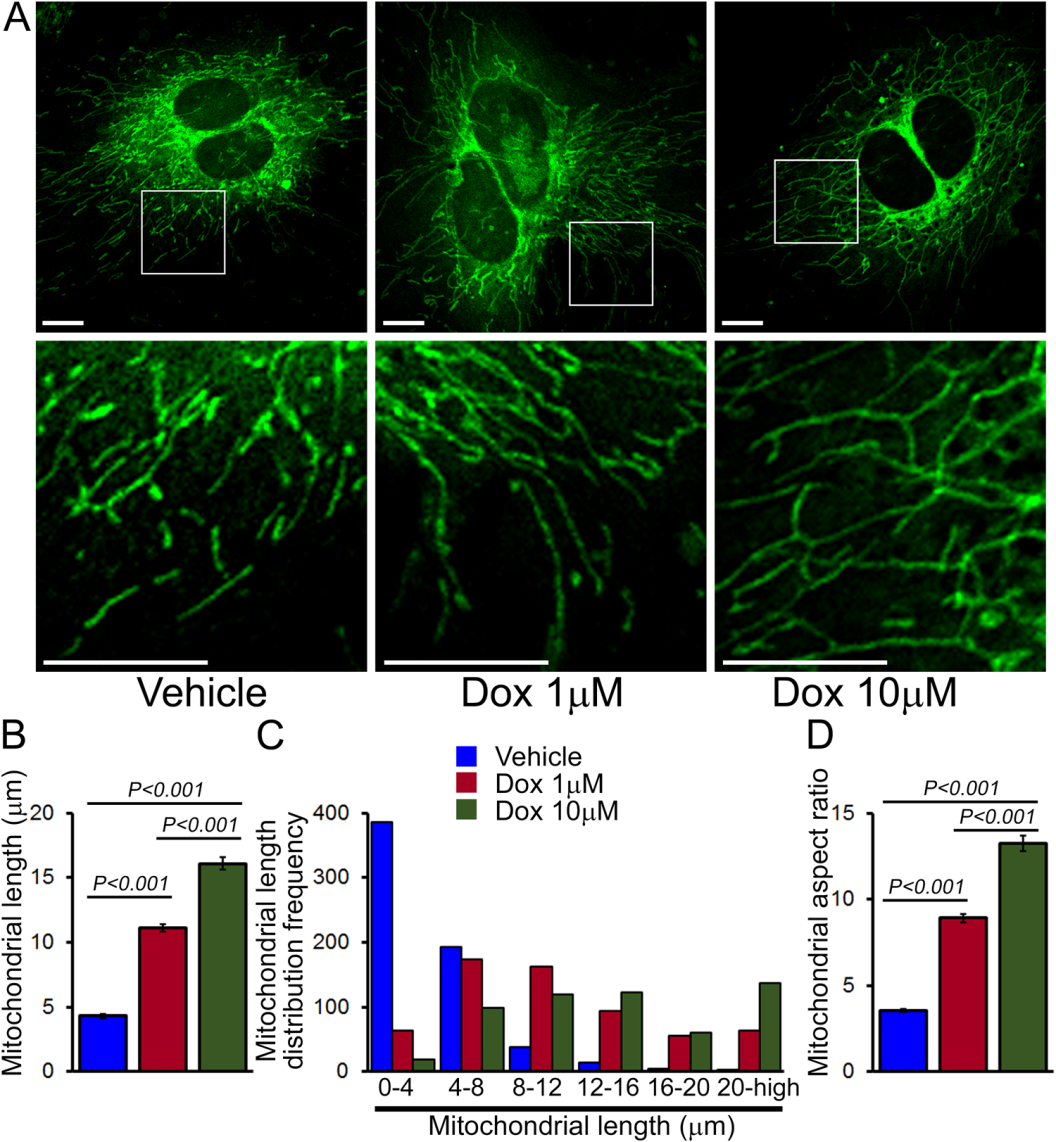
Altered mitochondrial morphology by Dox-treatment in cultured cardiomyocytes. (**A**) Representative images showing MitoTracker Green staining of mitochondria in NRCs treated with 1 μM and 10 μM Dox for 24 hours. Scale bar, 10 μm. Quantitative data for (**B**) mitochondrial length, (**C**) mitochondrial size distribution frequency and (**D**) mitochondrial aspect ratio. Bars represent mean ± SEM. *P* values were determined by Tukey’s *post-hoc* test.

To visualize the effect of Dox-treatment on mitochondrial morphology, NRCs were treated with Dox (1 µM and 10 µM) and vehicle for 24 hours. Confocal fluorescence studies of Dox-treated cardiomyocytes stained with MitoTracker Green showed increased mitochondrial hyperfusion (Fig. 11A) with increased mitochondrial length (Fig. 11B), mitochondrial length frequency distribution showing an increased number of large size mitochondria (Fig. 11C) and, increased mitochondrial organelle aspect ratio (Fig. 11D). We were able to demonstrate that Dox-treatment in NRCs significantly impaired autophagy activity, induced mitochondrial respiratory dysfunction, and altered mitochondrial dynamics recapitulating the *in vivo* Dox-cardiomyopathy data.

## Discussion

Dox-cardiotoxicity can manifest acutely as well as years after discontinuation of treatment leading to ventricular dysfunction, dilated cardiomyopathy and heart failure^31–33^. Our preclinical mouse model of acute and chronic Dox-treatment showed a gradual development of cardiomyopathy resulting in increased mortality. Both acute and chronic Dox-cardiomyopathy models showed increased accumulation of autophagosomes, altered expression of mitochondrial dynamics and oxidative respiration regulatory protein expression, and development of defects in mitochondrial respiration. We were able to recapitulate the *in vivo* autophagy and mitochondrial dysfunction data *in vitro* using cultured neonatal cardiomyocytes. Overall, our *in vivo* acute and chronic temporal study showed a direct association between autophagosomes accumulation and defects in mitochondrial respiration to the development of Dox-associated cardiomyopathy.

Autophagy is a dynamic cellular homeostasis process comprising of multiple steps including autophagosome biogenesis, autophagosome maturation, autophagosome fusion/docking with lysosome to form autolysosome and autolysosomal degradation^34–36^. Alterations of any of these steps cause impairment of cellular autophagy flux. Several studies have addressed the possible involvement of autophagy on the progression and development of Dox cardiomyopathy, but their findings were conflicting^7,8^. Both *in vivo* and *in vitro* analyses showed Dox-cardiotoxicity results in either increased^13–15^ or decreased^9–12^ autophagy activity. To resolve these discrepancies, we used biochemical LC3BII turn over assay, genetic reporter GFP-LC3 Tg mouse (monitor autophagosome) and tf-LC3 Tg mouse (monitor both autophagosome and autolysosome) to examine Dox-mediated alterations in different stages of autophagy process. Our temporal study using both acute and chronic Dox-cardiomyopathy models showed a gradual accumulation of autophagosomes resulting from impairment of the autophagic degradation process. We confirmed the biochemical data of Dox-induced autophagosomes accumulation *in vivo* by using GFP-LC3 and tf-LC3 reporter Tg mice. Recently, an elegant study also showed that chronic Dox-treatment blocks cardiomyocyte autophagic flux *in vivo* and in cultured cardiomyocytes which was accompanied by robust accumulation of undegraded autolysosomes^12^. We found the accumulation of LC3B II starts after 3d following acute Dox-treatment and 4 weeks in the chronic Dox-cardiomyopathy model. In contrast, Dox-treatment in neonatal cardiomyocytes showed a decreased level of LC3B II expression but at high doses of Dox- results in LC3B II accumulation. Despite the differences in steady-state LC3B ΙΙ expression levels in different Dox-treatment models (both *in vivo* and *in vitro*), our autophagy flux data suggest that Dox-treatment impairs autophagy flux resulting in the accumulation of autophagosomes and autolysosomes as well as defective autophagosome/autolysosome fusion. At present, we do not know whether suppression of either upstream or downstream steps of the autophagy process contributing to these impairment of autophagy flux. Our future studies are aimed to activate both the upstream and downstream autophagy signaling pathway to dissect the mechanism of Dox-mediated inhibition of autophagy flux.

We found a positive correlation with autophagosomes/autolysosomes accumulation and the development of systolic cardiac dysfunction, but we still do not know if autophagy is beneficial or detrimental in Dox-associated cardiotoxicity. Several studies have reported a beneficial effect of autophagy activation during Dox-cardiotoxicity; increasing autophagy in mice by either rapamycin or pre-fasting blunts Dox-induced cardiac dysfunction^10,11^. Similarly, treatment with 3-methyladenine was reported to preserve cardiac function after exposure to Dox^37^. Surprisingly, enhanced initiation of autophagy by over-expressing Beclin 1 Tg mice manifested an amplified cardiotoxic response, and Beclin 1 heterozygous deletion mice (*Beclin 1*^+/−^) showed protection from myocardial structural and functional changes in the chronic Dox cardiomyopathy model^12^. The conventional belief of autophagy activation by Beclin 1 overexpression was challenged by a study showing increased levels of Beclin 1 suppresses autophagosomes maturation leading to impaired, rather than increased autophagy flux^38,39^. Beclin 1 haploinsufficiency (as in Beclin 1^+/^) also increased autophagosome processing, i.e., increased autophagic flux^38^. Moreover, Beclin1 acts as a key scaffold for the assembly of distinct signaling complexes and performs important functions in multiple cellular processes including apoptosis, autophagy, endocytosis, phagocytosis, and cytokinesis^40,41^ and acts as tumor suppressor protein^42,43^. The possibility that Beclin 1 has additional autophagy-independent functions in cells is also supported by the embryonic lethal phenotype observed in *Beclin 1*^−/−^ mice^43^. In contrast, deletion of autophagy genes, *Atg5* or *Atg7*, does not result in lethality during embryogenesis^44,45^. All these discrepancies between studies on the role of autophagy in cardiac injury results from the use of non-specific autophagy activators and animal models. Therefore, our future studies with specific autophagy activation will allow us to resolve the discrepancies among these various studies and help us to define the consequences of autophagy activation (acute and chronic) in Dox-associated cardiomyopathy.

Mitochondrial dynamics are tightly regulated and are indispensable to maintain the normal morphology of the mitochondrial network to meet the metabolic needs of the cell. Mitochondrial fission is regulated and maintained by the dynamin-related GTPase Drp1. We found that only chronic Dox-treatment significantly increased DRP1 expression levels starting 4 weeks after Dox-exposure. Conversely, mitochondrial fusion is regulated by OPA1 and MFN2 proteins^46^. We also observed increased expression of OPA1 in both acute and chronic Dox-treated hearts. Chronic Dox-treatment showed decreased MFN2 expression; however expression was increased in the acute Dox-treated hearts. Overall, both acute and chronic Dox-treatment results in altered expression of mitochondrial dynamics regulatory protein expression. Mitochondrial morphology showed Dox-treatment induces mitochondrial hyperfusion with increased mitochondrial length and organelle aspect ratio. Alterations in mitochondrial dynamics by deletion of the fusion proteins Mfn1/Mfn2 or loss of OPA1 showed reduced activity of all respiratory complexes resulting in severe cellular defects^47,48^. Both acute and chronic-Dox cardiomyopathy models are associated with changes in OXPHOS and PDH protein expression. Mitochondria isolated from both acute and chronic Dox-treated hearts as well as intact neonatal cardiomyocytes showed suppression of mitochondrial bioenergetics. The Dox-treated hearts showed the imbalance of mitochondrial dynamics and impairment of mitochondrial bioenergetics. Reduction in mitochondrial OCR in Dox heart mitochondria could result from defects in mitochondrial substrate uptake, the flux through the electron transport chain, PDH activity, or the activity of the entire TCA cycle. Therefore, further studies are needed to define the role of Dox-cardiotoxicity on cardiac mitochondrial substrate preference.

Taken together, our data suggest that Dox-cardiomyopathy results from autophagosomes and autolysosomes accumulation, altered expression of mitochondrial dynamics and oxidative phosphorylation regulatory proteins and mitochondrial respiratory dysfunction. Previous studies of Dox-cardiotoxicity in animal models have maintained a narrow focus on either autophagy or mitochondria dysfunction to determine the causality of impairment in cardiomyocyte contractility. Our data show that increased autophagosomes accumulation due to inhibition of the autophagic degradation process and defects in mitochondrial respiration contribute to Dox-associated cardiomyopathy. More recent studies have shown that autophagy is essential in cellular metabolic and mitochondrial homeostasis^49^ and defects in autophagy result in mitochondrial dysfunction^50,51^. Future studies are needed to define whether impaired autophagic activity creates an imbalance in mitochondrial dynamics and defects in mitochondrial respiration resulting in Dox-cardiomyopathy. Moreover, given the critical role of mitochondria in the heart, it is very likely that altered mitochondrial dynamics and function may be a common pathway leading to cellular dysfunction critical to various cardiac diseases. Overall, our data indicate that activation of autophagy degradation process and restoration of mitochondrial function may be a valuable therapeutic target of Dox-toxicity.

## Materials and Methods

### Materials

Materials are as follows: DMEM (Gibco), FBS (Gibco), Cell Lytic M (Sigma-Aldrich), Protease Inhibitor Cocktail (Roche), pre-cast 7.5–15% Criterion Gels (BioRad), DAPI (Invitrogen), Doxorubicin (TSZ Chem, Biotang Inc.), Chloroquine (Sigma-Aldrich), Oligomycin (Sigma-Aldrich), FCCP (Sigma-Aldrich), Rotenone (Sigma-Aldrich), Antimycin A (Sigma-Aldrich), Ponceau S (Acros Organic), Vectashield Hardset (Vector Labs, H1400), and antimycin A (Sigma-Aldrich) were used.

### Animals

FVB/N mice of 8 to 10-weeks of age comprising both male and female of same litter were randomly assigned to study group. For the acute Dox-model, FVB/N mice were treated with a single intraperitoneal injection (i.p.) of Dox (20 mg/kg) or vehicle (saline) (Fig. 1A). For the chronic Dox-model FVB/N mice were subjected to four serial Dox (5 mg/kg) or vehicle injections weekly (Fig. 5A). Autophagy reporter mice GFP-LC3 Tg^19,21^ and mRFP-GFP-LC3 (tf-LC3)^19,23^ Tg mice are on the FVB/N genetic background. Mice used in the present studies were handled and cared according to the ‘Guide for the Care and Use of Laboratory Animals’ (National Institutes of Health, Bethesda, MD). All procedures involving research animals were approved by the ACUC Committee of LSU Health Sciences Center-Shreveport. Timed pregnant Sprague-Dawley rats were acquired from Charles River Laboratories International, Inc. (Portage, MI) for isolation of neonatal cardiomyocytes from newborn rat pups.

### Neonatal rat cardiomyocyte (NRC) isolation and culture

NRCs were isolated from the ventricles of 1–2-day old Sprague-Dawley rat pups as previously described^52,53^. Ventricular tissues collected from rat pups were digested with collagenase at 37 °C overnight and further digested in trypsin. After a preplating step to remove cardiac fibroblasts, isolated cardiomyocytes were plated at 1.5 × 10^6^ cells per 10-cm^2^ plate in αMEM containing 10% FBS (Gibco) and 1% antibiotic-antimycotic (Gibco). Cells underwent different treatments 24 h after plating and were maintained in DMEM (Gibco) containing 2% FBS and 1% antibiotic-antimycotic. All cell culture treatments were repeated in six independent experiments.

### Echocardiography

Echocardiograms were performed on isoflurane-anesthetized mice with a VisualSonics Vevo 3100 Imaging System (Toronto, Ontario, Canada) using a 40-MHz transducer to assess cardiac functional parameters^19,52,54^. Briefly, 2D directed M-mode echocardiographic images along the parasternal short axis were recorded by investigators blinded to genotype to determine LV size and systolic function. M-mode measurements included the LV internal dimensions in systole and diastole (LVIDs and LVIDd, respectively) as well as the diastolic thickness of LV posterior wall (LVPWd) and diastolic intraventricular septum thickness (IVSd). Percent fractional shortening (%FS) was calculated using: [(LVIDd − LVIDs)/LVIDd] × 100. From these data, LV end-systolic, LV end-diastolic diameter, LV mass and percent ejection fraction (%EF) were calculated.

### Mitochondria isolation

Mitochondria from Dox- and vehicle-treated hearts were isolated as described previously^52,55–57^. Briefly, hearts were harvested, and homogenized in MS-EGTA buffer (225 mM mannitol, 75 mM sucrose, 5 mM Hepes, and 1 mM EGTA, pH 7.4) and subjected to differential centrifugation. Finally, mitochondria were lysed with 1x Cell Lytic M (Sigma-Aldrich) containing protease and phosphatase inhibitors.

### Mitochondrial respiration

Mitochondrial oxygen consumption rate was measured with an XF24 Extracellular Flux Analyzer (Seahorse Biosciences, North Billerica MA) by methods as described previously^52,55–58^. Heart mitochondria were isolated using MS-EGTA buffer (225 mM mannitol, 75 mM sucrose, 5 mM Hepes, and 1 mM EGTA, pH 7.4) by differential centrifugation as described above. Mitochondria (50 μg/well) were seeded in XF24 culture plates, and respiration was measured in mitochondrial assay buffer (220 mM mannitol, 70 mM sucrose, 10 mM KH_2_PO_4_, 5 mM MgCl_2_, 2 mM HEPES, 1 mM EGTA, 0.2% fatty acid-free bovine serum albumin, pH 7.4) supplemented with 7 mM pyruvate and 1 mM malate. Mitochondrial oxygen consumption rate (OCR) was measured and plotted at basal conditions followed by sequential addition of 1 μg/ml oligomycin (ATP-synthase inhibitor), 4 μM FCCP (a mitochondrial uncoupler), and 0.5 μM rotenone (complex I inhibitor) plus 0.5 μM antimycin A (complex III inhibitor). The OCR values were normalized to total protein content in the corresponding wells and expressed as pmol/min/µg protein^52^.

For neonatal cardiomyocytes, NRCs were seeded at a density of 8 × 10^4^ cells/well into gelatin-coated Seahorse Bioscience XF microplates and were grown in DMEM (Gibco) containing 2% FBS (Gibco) and 1% antibiotic-antimycotic (Gibco)^52^. 48 h after plating the NRCs, cardiomyocytes were treated with different doses of Dox (1–25) for 6 hours and then measured the mitochondrial respiration. NRCs were incubated with DMEM (containing no glucose and pyruvate, Gibco) supplemented with 10 mM glucose and 2 mM pyruvate in a CO_2_-free incubator at 37 °C for 1 h before loading the plate in the XF24 analyzer. The OCR was measured over a period of 86 min over which time oligomycin (1 μM), FCCP (4 μM), and rotenone (0.5 μM) plus antimycin A (0.5 μM) were sequentially added to each well at specified time points.

### Autophagic flux

For the assessment of autophagic flux in the heart, mice were subjected to intraperitoneal injection with chloroquine diphosphate (50 mg/kg body weight; Sigma-Aldrich) as described previously^19^. Hearts were harvested, and LC3B II protein levels were determined by Western blot analysis. For *in vivo* determination of autophagic flux, GFP-LC3 Tg and tf-LC3 Tg mice were treated with Dox- and vehicle as mentioned in the acute (Fig. 1A) and chronic (Fig. 4A) Dox-treatment protocol and hearts were collected as different time points. To avoid autophagic induction during sample collection, hearts were perfused with 4% paraformaldehyde in cardioplegic buffer (0.1 M PBS, pH 7.4, 50–100 mM KCl, and 5% dextrose). Tissues were harvested and further fixed with 4% paraformaldehyde in PBS overnight (7–12 hours), followed by treatment with 15% sucrose in PBS for 4 hours and then with 30% sucrose solution overnight^19^. Tissue samples were embedded in Tissue-Tek OCT (Sakura Finetechnical Co. Ltd.) and stored at −80 °C. Frozen tissue samples were then sectioned at 5 μm thickness, air dried for 30 minutes, rehydrated in PBS for 5 minutes, mounted using a reagent containing DAPI, and viewed under a fluorescence microscope. Total numbers of GFP-LC3 and mRFP-LC3 dots in heart tissue sections were counted in individual green and red color channel confocal images in 10 high magnification fields for each heart sections in NIS Elements software (v4.13.04) and dots per cell were analyzed for each group of hearts as reported earlier^19^.

### Western blot analyses

Heart tissue lysates were prepared from hearts harvested in ice-cold PBS, pH 7.4 containing 1% Triton-X100, 2.5 mM EDTA, 0.5 mM PMSF, and a complete protease inhibitor mixture as described previously^52^. The heart homogenates were centrifuged at 12,000 × g for 15 min to sediment insoluble materials and the supernatants were collected for subsequent western blot experiments with appropriate antibodies.

Total proteins were extracted from NRCs washed with phosphate-buffered saline (PBS) and lysed with Cell Lytic M (Sigma-Aldrich) lysis buffer, supplemented with Complete Protease Inhibitor Cocktail (Roche)^1,52,53^. The lysed cells were homogenized by sonication and centrifuged at 14,000 × *g* for 15 min to sediment any insoluble material. The protein content of the soluble heart tissue and cardiomyocytes lysates were measured using the modified Bradford protocol/reagent relative to a BSA standard curve (BioRad). Protein lysates were separated on SDS-PAGE using pre-cast 7.5–15% Criterion Gels (BioRad) and transferred to PVDF membranes (BioRad). Membranes were blocked for 1 hr in 5% non-fat dried milk and exposed to primary antibodies overnight. The following primary antibodies were used for immunoblotting: Anti-Drp1 (1:1000, 14647, Cell Signaling Technology), Anti-Mfn2 (1:1000, 9482, Cell Signaling Technology), anti-OPA1 (1:1000, 80471, Cell Signaling Technology), Anti-β-Actin(1:1000, sc-47778, Santa Cruz Biotechnology), Anti-OXPHOS (1:1000, ab110413, Abcam), Anti-PDH (1:1000, ab110416, Abcam), Anti-LC3B (1:1000, 2775, Cell Signaling Technology), Anti-LAMP2 (1:1000, PA1–655, Invitrogen) and Anti-Cathepsin D (1:1000, sc-6487, Santa Cruz Biotechnology). Membranes were then washed, incubated with alkaline-phosphatase-conjugated secondary antibodies (Santa Cruz Biotechnology), developed with ECF reagent (Amersham) and imaged by using ChemiDoc™ Touch Imaging System (BioRad). Ponceau S protein staining on the transfer membrane was used as a loading control. Protein bands densitometry analysis on scanned membrane images was carried out using ImageJ software (NIH, Bethesda, MD).

### Mitochondria staining

To visualize mitochondria in cardiomyocytes, NRCs were plated on Lab-Tek II chamber slides (Thermo Scientific, 154461) at a density of 1 × 10^5^ cells/well as reported earlier^52,53^. Next, NRCs were treated with Doxorubicin (dissolved in DMSO) at 1 µM and 10 µM for 24 hours. NRCs treated with only DMSO served as experimental control cardiomyocytes. After 24 hours, NRCs were loaded with mitochondrial membrane potential independent MitoTracker Green FM (M7514, Molecular Probes, Invitrogen) at concentration of 200 nM dissolved in NRCs culture medium (2% FBS, 1% antibiotic in DMEM) for 40 minutes following immediate fixation with 3.7% paraformaldehyde in 1x PBS for 10 minutes as per manufacturer’s instructions (Molecular Probes, Invitrogen). After fixation, cells were washed with 1x PBS twice for 5 minutes each followed by mounting with Vectashield HardSet antifade mounting media for fluorescence (Vector Laboratories). Cardiomyocytes were subsequently observed on a Nikon A1R high-speed confocal microscope using an x60 oil objective (NA = 1.4) and imaged using Nikon NIS-Elements C software. To measure mitochondrial length (µm) and width (µm), mitochondria (>550 mitochondria for each treatment groups) from two independent experiments were analyzed on >15 high magnification field images in ≥50 cardiomyocytes by using NIH ImageJ (v1.6.0) software as described earlier^59,60^. Mitochondrial aspect ratio was calculated from the ratio of length and width as described^59,60^. All image analyses were performed in an investigator-blinded manner.

### Statistics

Data are expressed as mean ± SEM. All statistical tests were analyzed with GraphPad Prism software. Data were analyzed using Student’s *t*-test (*P* < 0.05) for two groups and groups of four or more with one-way ANOVA, followed by Tukey’s *post hoc* test.

## Supplementary information

Supplement figures

## Data Availability

All Western Blot raw data generated or analyzed during this study are included in this published article. Other datasets generated during and/or analyzed during the current study are available from the corresponding author on reasonable request.

## References

[1] Singal PK, Iliskovic N (1998). Doxorubicin-induced cardiomyopathy. N Engl J Med.

[2] Singal PK, Li T, Kumar D, Danelisen I, Iliskovic N (2000). Adriamycin-induced heart failure: mechanism and modulation. Mol Cell Biochem.

[3] Middleman E, Luce J, Frei E (1971). Clinical trials with adriamycin. Cancer.

[4] Ichikawa Y (2014). Cardiotoxicity of doxorubicin is mediated through mitochondrial iron accumulation. J Clin Invest.

[5] Sawyer DB (2013). Anthracyclines and heart failure. N Engl J Med.

[6] Simunek T (2009). Anthracycline-induced cardiotoxicity: overview of studies examining the roles of oxidative stress and free cellular iron. Pharmacol Rep.

[7] Koleini N, Kardami E (2017). Autophagy and mitophagy in the context of doxorubicin-induced cardiotoxicity. Oncotarget.

[8] Bartlett JJ, Trivedi PC, Pulinilkunnil T (2017). Autophagic dysregulation in doxorubicin cardiomyopathy. J Mol Cell Cardiol.

[9] Li S (2014). Nrf2 deficiency exaggerates doxorubicin-induced cardiotoxicity and cardiac dysfunction. Oxid Med Cell Longev.

[10] Kawaguchi T (2012). Prior starvation mitigates acute doxorubicin cardiotoxicity through restoration of autophagy in affected cardiomyocytes. Cardiovasc Res.

[11] Sishi BJ, Loos B, van Rooyen J, Engelbrecht AM (2013). Autophagy upregulation promotes survival and attenuates doxorubicin-induced cardiotoxicity. Biochem Pharmacol.

[12] Li DL (2016). Doxorubicin Blocks Cardiomyocyte Autophagic Flux by Inhibiting Lysosome Acidification. Circulation.

[13] Kobayashi S (2010). Transcription factor GATA4 inhibits doxorubicin-induced autophagy and cardiomyocyte death. J Biol Chem.

[14] Dhingra R (2014). Bnip3 mediates doxorubicin-induced cardiac myocyte necrosis and mortality through changes in mitochondrial signaling. Proc Natl Acad Sci USA.

[15] Wang X (2014). Ghrelin inhibits doxorubicin cardiotoxicity by inhibiting excessive autophagy through AMPK and p38-MAPK. Biochem Pharmacol.

[16] Zhu W (2009). Acute doxorubicin cardiotoxicity is associated with p53-induced inhibition of the mammalian target of rapamycin pathway. Circulation.

[17] Walker JR (2011). The cardioprotective role of probucol against anthracycline and trastuzumab-mediated cardiotoxicity. J Am Soc Echocardiogr.

[18] Mizushima N, Yoshimori T (2007). How to interpret LC3 immunoblotting. Autophagy.

[19] Bhuiyan MS (2013). Enhanced autophagy ameliorates cardiac proteinopathy. J Clin Invest.

[20] McLendon PM (2014). Tubulin hyperacetylation is adaptive in cardiac proteotoxicity by promoting autophagy. Proc Natl Acad Sci USA.

[21] Zhu H (2007). Cardiac autophagy is a maladaptive response to hemodynamic stress. J Clin Invest.

[22] Kimura S, Noda T, Yoshimori T (2007). Dissection of the autophagosome maturation process by a novel reporter protein, tandem fluorescent-tagged LC3. Autophagy.

[23] Hariharan N, Zhai P, Sadoshima J (2011). Oxidative stress stimulates autophagic flux during ischemia/reperfusion. Antioxid Redox Signal.

[24] Homewood CA, Warhurst DC, Peters W, Baggaley VC (1972). Lysosomes, pH and the anti-malarial action of chloroquine. Nature.

[25] Dott W, Mistry P, Wright J, Cain K, Herbert KE (2014). Modulation of mitochondrial bioenergetics in a skeletal muscle cell line model of mitochondrial toxicity. Redox Biol.

[26] Hill BG (2012). Integration of cellular bioenergetics with mitochondrial quality control and autophagy. Biol Chem.

[27] Brand MD, Nicholls DG (2011). Assessing mitochondrial dysfunction in cells. Biochem J.

[28] Gianni L (1997). Human pharmacokinetic characterization and *in vitro* study of the interaction between doxorubicin and paclitaxel in patients with breast cancer. J Clin Oncol.

[29] Gustafson DL, Rastatter JC, Colombo T, Long ME (2002). Doxorubicin pharmacokinetics: Macromolecule binding, metabolism, and excretion in the context of a physiologic model. J Pharm Sci.

[30] van Asperen J, van Tellingen O, Tijssen F, Schinkel AH, Beijnen JH (1999). Increased accumulation of doxorubicin and doxorubicinol in cardiac tissue of mice lacking mdr1a P-glycoprotein. Br J Cancer.

[31] Colombo A, Cipolla C, Beggiato M, Cardinale D (2013). Cardiac toxicity of anticancer agents. Curr Cardiol Rep.

[32] Lipshultz SE (2012). Changes in cardiac biomarkers during doxorubicin treatment of pediatric patients with high-risk acute lymphoblastic leukemia: associations with long-term echocardiographic outcomes. J Clin Oncol.

[33] Lipshultz SE (2010). Assessment of dexrazoxane as a cardioprotectant in doxorubicin-treated children with high-risk acute lymphoblastic leukaemia: long-term follow-up of a prospective, randomised, multicentre trial. Lancet Oncol.

[34] Bento CF (2016). Mammalian Autophagy: How Does It Work?. Annu Rev Biochem.

[35] Klionsky DJ (2016). Guidelines for the use and interpretation of assays for monitoring autophagy (3rd edition). Autophagy.

[36] Mizushima N, Yoshimori T, Levine B (2010). Methods in mammalian autophagy research. Cell.

[37] Lu L (2009). Adriamycin-induced autophagic cardiomyocyte death plays a pathogenic role in a rat model of heart failure. Int J Cardiol.

[38] Ma X (2012). Impaired autophagosome clearance contributes to cardiomyocyte death in ischemia/reperfusion injury. Circulation.

[39] Jimenez RE, Kubli DA, Gustafsson AB (2014). Autophagy and mitophagy in the myocardium: therapeutic potential and concerns. Br J Pharmacol.

[40] Wirawan E (2012). Beclin1: a role in membrane dynamics and beyond. Autophagy.

[41] Nikoletopoulou V, Markaki M, Palikaras K, Tavernarakis N (2013). Crosstalk between apoptosis, necrosis and autophagy. Biochim Biophys Acta.

[42] Qu X (2003). Promotion of tumorigenesis by heterozygous disruption of the beclin 1 autophagy gene. J Clin Invest.

[43] Yue Z, Jin S, Yang C, Levine AJ, Heintz N (2003). Beclin 1, an autophagy gene essential for early embryonic development, is a haploinsufficient tumor suppressor. Proc Natl Acad Sci USA.

[44] Komatsu M (2005). Impairment of starvation-induced and constitutive autophagy in Atg7-deficient mice. J Cell Biol.

[45] Kuma A (2004). The role of autophagy during the early neonatal starvation period. Nature.

[46] Wai T, Langer T (2016). Mitochondrial Dynamics and Metabolic Regulation. Trends Endocrinol Metab.

[47] Chen H, Chomyn A, Chan DC (2005). Disruption of fusion results in mitochondrial heterogeneity and dysfunction. J Biol Chem.

[48] Muster B (2010). Respiratory chain complexes in dynamic mitochondria display a patchy distribution in life cells. PLoS One.

[49] Kawakami T (2015). Deficient Autophagy Results in Mitochondrial Dysfunction and FSGS. J Am Soc Nephrol.

[50] Suzuki SW, Onodera J, Ohsumi Y (2011). Starvation induced cell death in autophagy-defective yeast mutants is caused by mitochondria dysfunction. PLoS One.

[51] Medeiros TC, Thomas RL, Ghillebert R, Graef M (2018). Autophagy balances mtDNA synthesis and degradation by DNA polymerase POLG during starvation. J Cell Biol.

[52] Alam, S. *et al*. Aberrant Mitochondrial Fission Is Maladaptive in Desmin Mutation-Induced Cardiac Proteotoxicity. *J Am Heart Assoc* **7**, 10.1161/JAHA.118.009289 (2018). 10.1161/JAHA.118.009289 PMC6064863 29987122

[53] Alam, S. *et al*. Sigmar1 regulates endoplasmic reticulum stress-induced C/EBP-homologous protein expression in cardiomyocytes. *Biosci Rep* **37**, 10.1042/BSR20170898 (2017). 10.1042/BSR20170898 PMC5518542 28667101

[54] Bhuiyan MS (2016). *In vivo* definition of cardiac myosin-binding protein C’s critical interactions with myosin. Pflugers Arch.

[55] Karch J (2013). Bax and Bak function as the outer membrane component of the mitochondrial permeability pore in regulating necrotic cell death in mice. Elife.

[56] Kwong JQ (2015). The Mitochondrial Calcium Uniporter Selectively Matches Metabolic Output to Acute Contractile Stress in the Heart. Cell Rep.

[57] Abdullah CS (2018). Cardiac Dysfunction in the Sigma 1 Receptor Knockout Mouse Associated With Impaired Mitochondrial Dynamics and Bioenergetics. J Am Heart Assoc.

[58] Chandra M (2018). Cardiac-specific inactivation of LPP3 in mice leads to myocardial dysfunction and heart failure. Redox Biol.

[59] Song M, Franco A, Fleischer JA, Zhang L, Dorn GW (2017). Abrogating Mitochondrial Dynamics in Mouse Hearts Accelerates Mitochondrial Senescence. Cell Metab.

[60] Song M, Mihara K, Chen Y, Scorrano L, Dorn GW (2015). Mitochondrial fission and fusion factors reciprocally orchestrate mitophagic culling in mouse hearts and cultured fibroblasts. Cell Metab.

