# Supplementary material for: Doxorubicin-induced cardiomyopathy associated with inhibition of autophagic degradation process and defects in mitochondrial respiration
Source: Sci Rep. 2019 Feb 14;9:2002. doi: 10.1038/s41598-018-37862-3 (PMC6376057; doi:10.1038/s41598-018-37862-3)
Supplement: Supplementary file 1 — Supplement figures [file 41598_2018_37862_MOESM1_ESM.pdf]

Doxorubicin-induced cardiomyopathy associated with inhibition of autophagic degradation process and defects in mitochondrial respiration

Chowdhury S Abdullah, PhD;[1] Shafiul Alam,PhD;[1] Richa Aishwarya, BS;[2] Sumitra Miriyala, PhD;[3] Mohammad Alfrad Nobel Bhuiyan, MS;[4] Manikandan Panchatcharam,PhD;[3] Christopher B. Pattillo, PhD;[2] A. Wayne Orr, PhD;[1,2,3] Junichi Sadoshima, MD, PhD;[5] Joseph A Hill, MD, PhD;[6] Md. Shenuarin Bhuiyan, PhD[1,2*]

[1]Department of Pathology and Translational Pathobiology, Louisiana State University Health Sciences Center-Shreveport, Shreveport, LA 71103, USA
[2]Department of Molecular and Cellular Physiology, Louisiana State University Health Sciences Center-Shreveport, Shreveport, LA 71103, USA
[3]Department of Cellular Biology and Anatomy, Louisiana State University Health Sciences Center-Shreveport, Shreveport, LA 71103, USA
[4]Division of Biostatistics and Epidemiology, Cincinnati Children's Hospital, Cincinnati, OH 45229, USA
[5]Department of Cell Biology and Molecular Medicine, Rutgers New Jersey Medical School, Newark, NJ 07103, USA
[6]Department of Internal Medicine (Cardiology), UT Southwestern Medical Center, and Department of Molecular Biology, UT Southwestern Medical Center, UT Southwestern Medical Center, Dallas, TX 75390, USA

[*]Corresponding author

* **Correspondence to:** Md. Shenuarin Bhuiyan, Department of Pathology and Translational Pathobiology & Department of Molecular and Cellular Physiology, Louisiana State University Health Sciences Center, Shreveport LA 71103, USA, Ph: 318-675-3369, Fax: 318-675-33; E-mail: mbhuiy@lsuhsc.edu

Pages: 24 Figures: 11 Supplement Figures: 3

Short Title: Pathogenic sequelae of Dox-cardiomyopathy
Conflicts of Interest: none
Author contributions: C.S.A., S.A., R.A., and M.S.B. performed experiments. S.M. analyzed data. M.A.N.B performed statistical analysis. A.W.O., C.B.P and M.P. contributed to analytic tools. J.S. and J.A.H contributed to reagents. C.S.A. and M. S. B. designed experiments. C.S.A. and M.S.B. wrote the manuscript and all authors contributed to the preparation of the manuscript.

Keywords: Doxorubicin, cardiomyopathy, autophagy, mitochondrial dynamics, mitochondrial respiration

Subject Categories: **Molecular Cardiology**

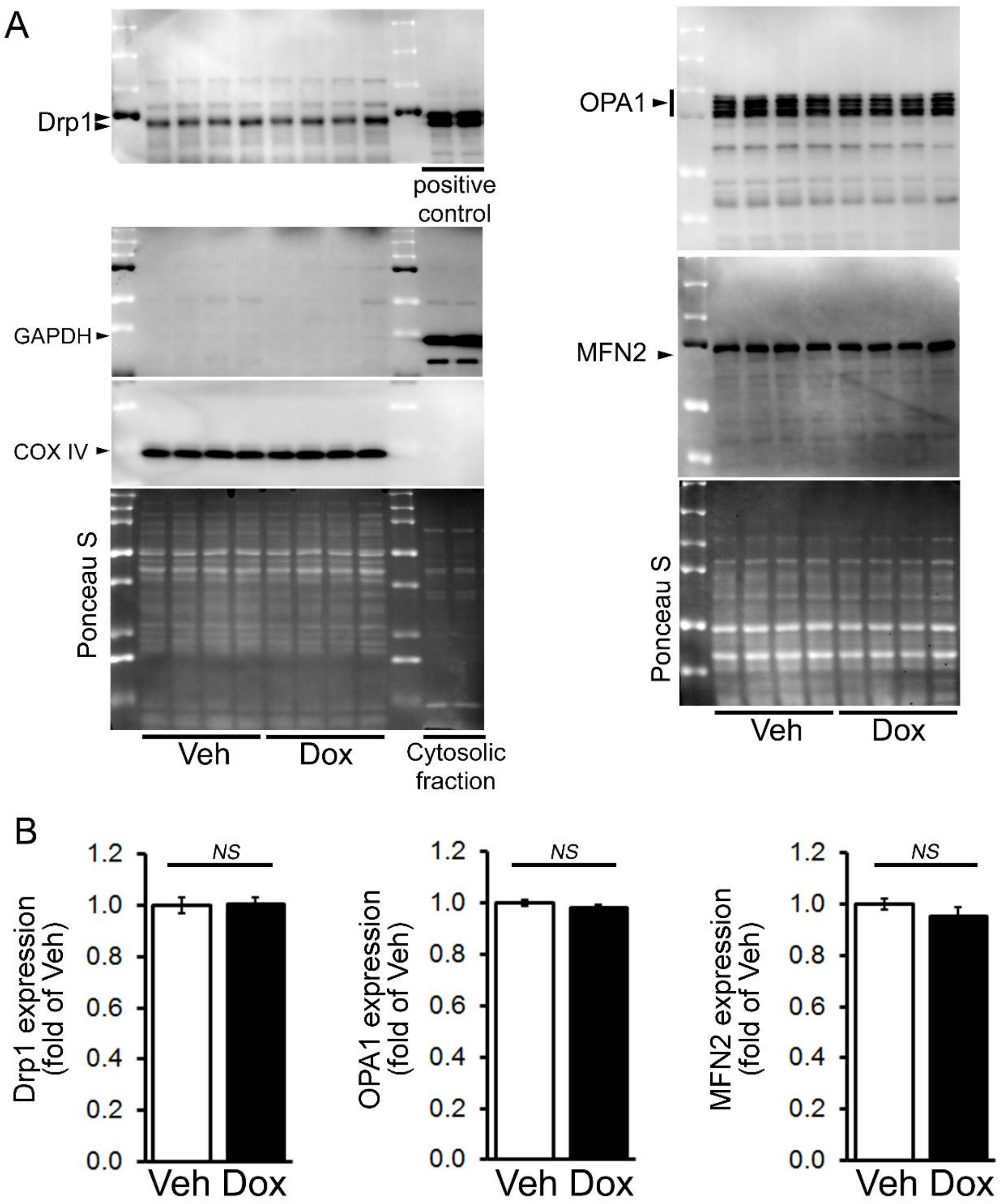

Supplement Figure S1. Expression of mitochondrial dynamic regulatory proteins in the mitochondrial fraction of acute Dox-treated mice. (**A**) Representative Western blot and (**B**) densitometric quantification of the expression of mitochondrial dynamic regulatory proteins in the mitochondrial fraction isolated from the hearts of 5 days after vehicle and acute Dox-treated mice: Drp1, OPA1, and MFN2. Drp1 overexpressing HEK293 cells were used as a positive control for Drp1. Cytosol fraction was used as a control to show the purity of the mitochondrial fraction and confirmed by GAPDH. COXIV was run on the same membrane to confirm mitochondrial extracts. Ponceau S protein staining of the transfer membrane confirmed approximately equal loading across the gel (n=4 mice per group). Bars represent mean ± SEM. n=4 mice per group at each time point. *P* values were determined by Tukey's *post-hoc* test. *NS* = not significant.

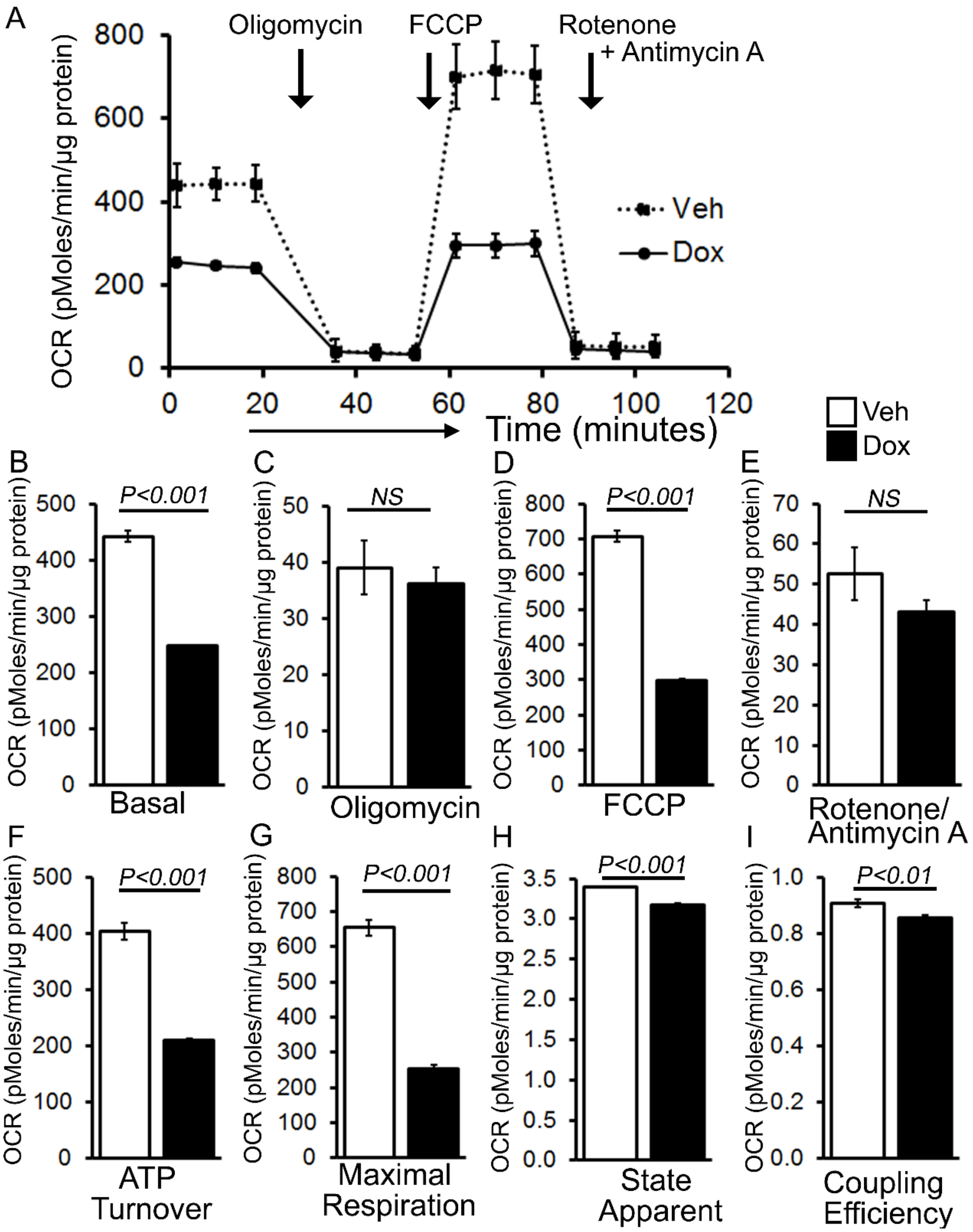

Supplement Figure S2. Suppression of mitochondrial respiration in the hearts of acute Dox-cardiomyopathy mice. (**A**) Mitochondrial oxygen consumption rate (OCR) profiles in isolated mitochondria from 5 days after acute Dox-treated hearts. Arrow indicates the sequential addition of oligomycin (1 µM), FCCP (4 µM), and rotenone (0.5 µM) plus antimycin A (0.5 µM). OCR profile is expressed as pMolesO_2/min/µg of protein. Graph showing OCR under (**B**) baseline as well as with the addition of (**C**) oligomycin, (**D**) FCCP, and (**E**) rotenone plus antimycin A. Key parameters of mitochondrial function, including (**F**) ATP turnover, (**G**) maximal respiration, (**H**) state apparent and (**I**) coupling efficiency were significantly decreased in Dox mice. Bars represent mean ± SEM. n=6 mice per group. *P* values were determined by Tukey's *post-hoc* test.

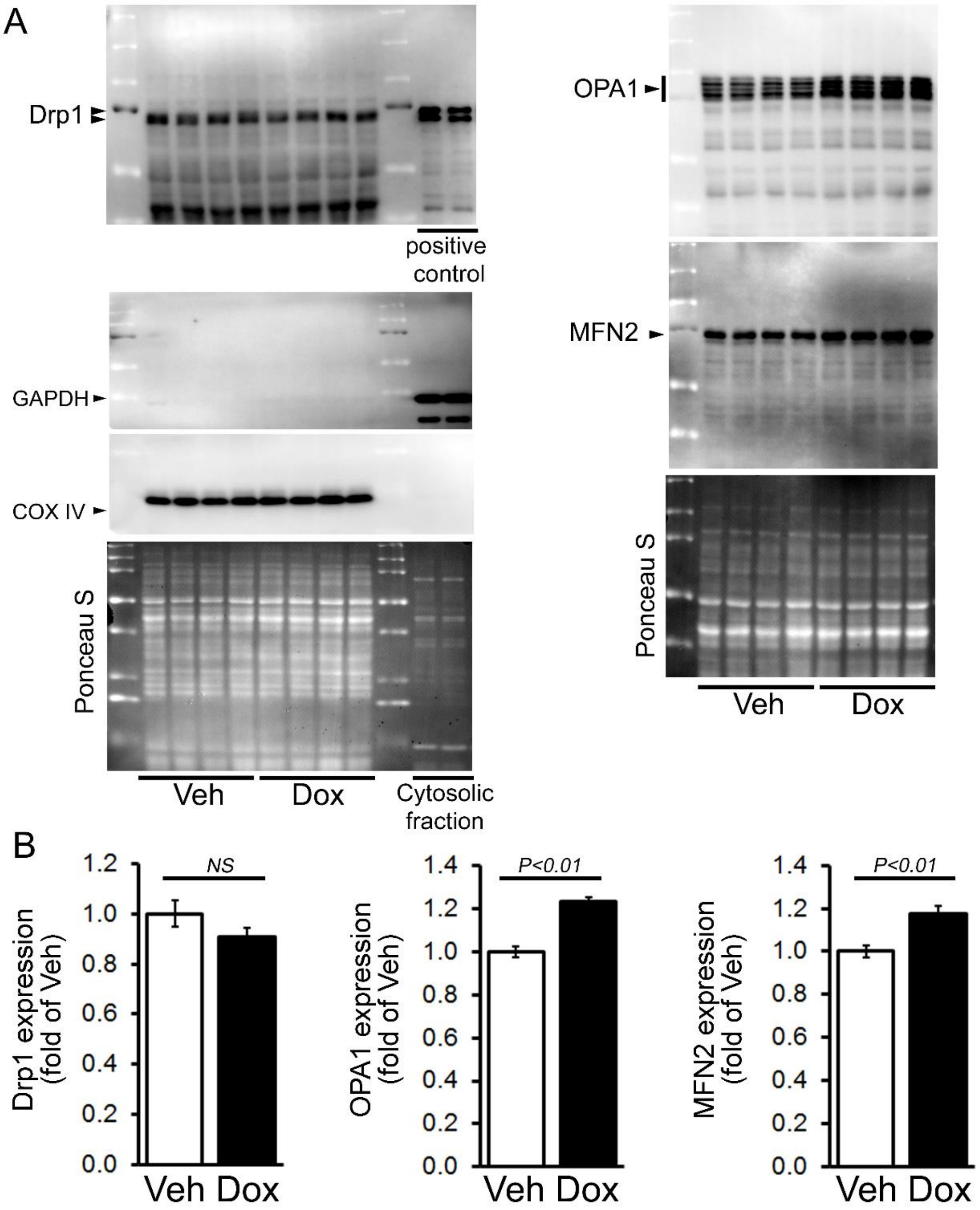

Supplement Figure S3. Expression of mitochondrial dynamic regulatory proteins in the mitochondrial fraction of chronic Dox-treated mice. (**A**) Representative Western blot and (**B**) densitometric quantification of the expression of mitochondrial dynamic regulatory proteins in the mitochondrial fraction isolated from the hearts of 12 weeks after vehicle and acute Dox-treated mice: Drp1, OPA1, and MFN2. Drp1 overexpressing HEK293 cells were used as a positive control for Drp1. Cytosol fraction was used as a control to show the purity of the mitochondrial fraction and confirmed by GAPDH. COXIV was run on the same membrane to confirm mitochondrial extracts. Ponceau S protein staining of the transfer membrane confirmed approximately equal loading across the gel (n=4 mice per group). Bars represent mean ± SEM. n=4 mice per group at each time point. *P* values were determined by Tukey's *post-hoc* test. *NS* = not significant.
